# Organic Electronics in Biosensing: A Promising Frontier for Medical and Environmental Applications

**DOI:** 10.3390/bios13110976

**Published:** 2023-11-07

**Authors:** Jyoti Bala Kaushal, Pratima Raut, Sanjay Kumar

**Affiliations:** 1 Department of Biochemistry and Molecular Biology, University of Nebraska Medical Center, Omaha, NE 68198, USA; jyoti.kaushal@unmc.edu (J.B.K.); pratima.raut@unmc.edu (P.R.); 2 Durham School of Architectural Engineering and Construction, Scott Campus, University of Nebraska-Lincoln, Omaha, NE 68182, USA

**Keywords:** organic electronics, OECT, OFET, OPD, implants, biosensors

## Abstract

The promising field of organic electronics has ushered in a new era of biosensing technology, thus offering a promising frontier for applications in both medical diagnostics and environmental monitoring. This review paper provides a comprehensive overview of organic electronics’ remarkable progress and potential in biosensing applications. It explores the multifaceted aspects of organic materials and devices, thereby highlighting their unique advantages, such as flexibility, biocompatibility, and low-cost fabrication. The paper delves into the diverse range of biosensors enabled by organic electronics, including electrochemical, optical, piezoelectric, and thermal sensors, thus showcasing their versatility in detecting biomolecules, pathogens, and environmental pollutants. Furthermore, integrating organic biosensors into wearable devices and the Internet of Things (IoT) ecosystem is discussed, wherein they offer real-time, remote, and personalized monitoring solutions. The review also addresses the current challenges and future prospects of organic biosensing, thus emphasizing the potential for breakthroughs in personalized medicine, environmental sustainability, and the advancement of human health and well-being.

## 1. Introduction

Organic electronics have emerged as a promising frontier in the field of biosensing, thereby offering innovative and versatile solutions for medical and environmental applications. With the rapid advancement of organic materials and devices, integrating organic electronics into biosensing platforms has unlocked many possibilities for sensitive, real-time, and label-free biological and chemical analyte detection. This convergence of organic electronics and biosensing can revolutionize medical diagnostics, point-of-care testing, wearable health monitoring, and environmental monitoring, among other critical domains.

Based on carbon-based compounds and polymers, organic electronic devices present distinct advantages that make them well-suited for biosensing applications. These materials offer biocompatibility, thereby enabling direct interactions with biological systems without causing adverse reactions, thus making them ideal for implantable biosensors and in vivo monitoring. Additionally, organic materials exhibit exceptional flexibility, thus enabling the development of conformable and wearable biosensing devices that can seamlessly adapt to the human body or environmental surfaces, thus expanding their utility in personalized healthcare and environmental monitoring. The unique electronic properties of organic materials, such as tunability, conductivity, and semiconducting behavior, contribute to their exceptional sensing capabilities. Organic electronic devices, such as organic field-effect transistors (OFETs), organic electrochemical transistors (OECTs), and organic photodetectors (OPDs), have demonstrated high sensitivity, selectivity, and rapid response times, thereby allowing for the accurate detection of target analytes in complex samples.

In this context, this review explores the exciting frontier of organic electronics in biosensing, wherein it focuses on its applications in medical diagnostics and environmental monitoring. We delve into an overview of organic bioelectronic materials, the various organic electronic devices, and their fabrication methods, thus detailing their sensing mechanisms and advantages over traditional sensing technologies. Additionally, we discuss the challenges faced in integrating organic electronics in biosensing platforms, such as biocompatibility, stability, manufacturing scalability, and data security and privacy, as well as the innovative strategies employed to address these obstacles.

## 2. Organic Bioelectronic Materials

### 2.1. Conducting Polymers

Conducting polymers (CPs), also known as intrinsically conducting polymers (ICPs) or conjugated polymers, are a class of organic materials that exhibit electrical conductivity while maintaining the desirable mechanical properties of polymers. Unlike traditional semiconductors, CPs are intrinsically conductive without requiring any additional dopants. This unique combination of electrical and mechanical properties makes conducting polymers highly attractive for various applications, including electronics, biosensors, actuators, and energy storage devices. The electrical conductivity of conducting polymers arises from the delocalization of the π electrons, which occurs through the presence of alternating single and double bonds along their polymer chains. These π electrons can move freely through the conjugated system, thereby allowing the movement of charge carriers (electrons and holes), which consequently results in electrical conductivity. CPs possess a valence band (HOMO—highest occupied molecular orbital) and a conduction band (LUMO—lowest unoccupied molecular orbital) [1]. The energy gap between the HOMO and the LUMO determines the material’s band gap, thereby affecting its electrical properties [2]. In their pure, undoped state, organic polymers may behave as insulators or semiconductors due to the large energy gap between the HOMO and the LUMO [3]. Nonetheless, doping allows these materials to become conductive. Doping entails the introduction of additional charge carriers into the material, which is achieved through the incorporation of electron donors (n-type doping) or electron acceptors (p-type doping). This deliberate addition of charge carriers reduces the energy gap, thereby facilitating the movement of charge carriers and enhancing the electrical conductivity of the organic polymers. However, it is important to emphasize that not all organic semiconductors require external doping to attain their intended electrical properties. Some organic semiconductors demonstrate inherent doping behavior, often referred to as self-doping, without the deliberate addition of external dopants. Notable examples include self-doped poly(3-alkylsulfonate pyrroles) and self-n-doped perylene diimides (PDIs) [4,5,6]. Within these organic semiconductors, self-doping typically arises due to the presence of impurities, defects, or structural anomalies within the material itself. These imperfections can introduce charge carriers into the energy band structure of the semiconductor, thus resulting in either p-type behavior (conduction dominated by holes) or n-type behavior (conduction dominated by electrons).

In the case of CPs, the energy gap (band gap) between the HOMO and the LUMO is relatively small compared to insulators but larger than for true metals. CPs exhibit distinct electrical and mechanical properties, thereby allowing researchers to tailor their performance for specific applications. The electrical conductivity of conducting polymers can be tuned according to various factors such as oxidation state, doping level, and environmental conditions. CPs can be chemically doped or electrochemically doped to enhance their conductivity. By doping, additional charge carriers are introduced into the material, thus increasing the electrical conductivity. Moreover, the mechanical properties of CPs are influenced by factors such as molecular weight, chemical structure, and processing methods. These polymers can be synthesized into various forms, including films, fibers, and coatings, all while retaining conductivity. The flexibility and ease of processability make conducting polymers suitable for applications where traditional inorganic conductors may be limited due to their rigidity. The unique combination of electrical conductivity and mechanical flexibility enables conducting polymers to be used in electronic devices such as organic transistors, flexible displays, and printed circuits. They are also employed as sensing elements in chemical sensors and biosensors, where their conductivity changes upon interaction with specific analytes. In the field of energy storage, conducting polymers have been explored for applications in supercapacitors and batteries due to their high charge-storage capacity.

**Figure 1:**
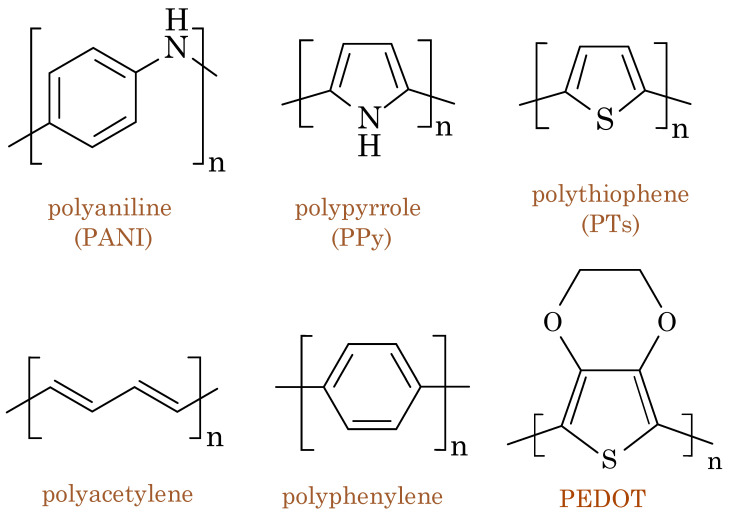
Chemical structures of commonly used conducting polymers: polyaniline (PANI), polypyrrole (PPy), polythiophene(PTs), polyacetylene, polyphenylene, and poly(3,4-ethylenedioxythiophene) (PEDOT).

One of the pioneering conducting polymers is polyaniline (PANI), which first was discovered for its conductive properties in the late 1970s. Since then, several other conducting polymers, such as polythiophene(PTs), polypyrrole (PPy), and poly(3,4-ethylenedioxythioph ene) (PEDOT), have been developed and extensively studied [7]. PEDOT is the most ubiquitous organic mixed ionic/electronic conductor (OMIEC); it constitutes a class of materials exhibiting simultaneous electronic and ionic conductivity [8]. This unique combination of properties makes OMIECs highly valuable for various applications, including electrochemical devices, energy storage systems, actuators, artificial muscles, and biosensors [9]. OMIECs comprise soft organic materials, such as conducting polymers or small organic molecules, that can conduct electrons and ions, thereby offering advantages over traditional electronic or ionic conductors [10]. Figure 1 shows the chemical structures of commonly used conducting polymers.

### 2.2. Organic Semiconductors

Organic semiconductors are a class of organic materials with unique electronic properties, thus lying between traditional conductors and insulators. The band gap between the organic semiconductors’ valence and conduction bands is relatively lower than in insulators and higher than in conducting polymers. The organic semiconductors are composed of carbon-based molecules or polymers, known as π-conjugated systems, which enable the movement of charge carriers (electrons and holes) through their conjugated molecular structures [11,12,13,14].

Small-molecule semiconductors consist of discrete, well-defined organic molecules, while polymer-based semiconductors comprise long-chain polymer structures with repeating monomer units. Organic molecules primarily comprise carbon atoms bonded to hydrogen, oxygen, nitrogen, and other elements. Carbon’s ability to form stable covalent bonds with various other atoms allows for the diverse and complex structures found in organic molecules. Organic molecules contain specific functional groups, which are arrangements of atoms that confer distinct chemical properties and reactivity to the molecule. For example, the hydroxyl group (-OH) in alcohols is hydrophilic, while the carbonyl group (-C=O) in ketones and aldehydes has unique reactivity. Key organic molecular semiconductors include oligoacenes, oligothiophenes, discotic liquid crystals, triphenylamines, perylenes, tetrathiafulvalenes, and fullerenes [15]. On the other hand, polymer-based semiconductors, similar to conducting polymers used in electronic devices, possess a well-defined energy band gap, thereby allowing them to transition between conductive and insulating states. Prominent examples of these organic polymeric semiconductors include polyparaphenylenevinylene (PPV), polyparaphenylene (PPP), polyfluorene (PF), and polyfluorene copolymers [16]. Both types of organic semiconductors offer numerous advantages, including solution processability and low-temperature deposition, thus rendering them suitable for a wide range of electronic and optoelectronic applications. Their versatility is pivotal for developing innovative and cost-effective devices for contemporary technologies.

**Figure 2:**
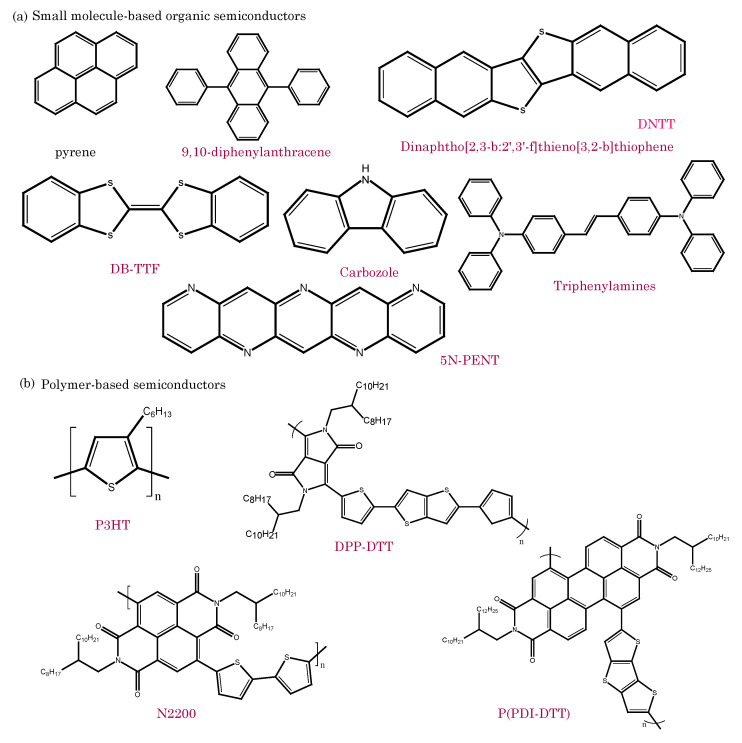
Chemical structures of organic semiconductors: (**a**) π-conjugated small molecular families based semiconductors and (**b**) polymer-based semiconductors.

The unique properties of these materials have led to their integration into a wide range of electronic devices, such as organic field-effect transistors (OFETs) [17,18], organic light-emitting diodes (OLEDs) [19,20,21], organic photovoltaics (OPVs) [22,23,24], and organic sensors [25]. OFETs utilize organic semiconductors as the active channel material, thereby enabling flexible and low-power transistor devices. Organic sensors utilize the sensitivity of organic semiconductors to detect changes in environmental parameters, such as gas concentration or biomolecular interactions [26]. Figure 2 shows examples of commonly used small-molecule-based and polymer-based organic semiconductors for different types of bioelectronics devices [27,28,29].

While organic semiconductors have numerous advantages, such as flexibility and cost-effectiveness, they are not without challenges. These challenges encompass relatively lower charge carrier mobility when compared to their inorganic counterparts, as well as a susceptibility to environmental factors like humidity and temperature, as noted in recent studies [30,31,32]. To overcome these limitations, researchers are actively exploring advanced material engineering, innovative doping techniques, and novel device architectures to enhance the performance and stability of organic semiconductors [33,34].

Organic semiconductors remain a highly promising platform for developing flexible, cost-effective, and energy-efficient electronic devices [35]. Their unique properties and versatile applications have positioned them as compelling candidates for the next generation of electronic and optoelectronic innovations. These advancements drive innovation across various domains, including wearable technology, flexible displays, and renewable energy solutions. As research and development in organic semiconductors continue to progress, new opportunities are anticipated to emerge in the ever-evolving realm of organic electronics.

### 2.3. Biomolecules as Sensing Elements

Biomolecules serve as highly sensitive and selective sensing elements in various biosensing applications. These natural macromolecules, including proteins, nucleic acids, enzymes, and antibodies, exhibit specific interactions with target analytes, thereby enabling the detection and quantification of various substances with remarkable accuracy. The inherent recognition capabilities of biomolecules make them valuable sensing elements in biosensors, thus enabling real-time monitoring of biochemical reactions and detecting of analytes with exceptional specificity.

One of the key advantages of using biomolecules as sensing elements is their ability to bind specifically to target molecules, known as ligands or antigens, through molecular recognition processes [36]. This binding interaction is governed by complementary shapes and chemical properties between the biomolecule’s active sites and the target analyte, thus allowing for highly selective detection [37]. The high affinity of biomolecules to their target analytes ensures that biosensors can distinguish between similar molecules, thus achieving precise and reliable measurements. Various techniques are employed to immobilize biomolecules onto the sensor surface while maintaining biological activity. Surface modification methods, such as physical adsorption, covalent binding, and self-assembled monolayers, allow the biomolecules to remain functional while attached to the sensor surface [38,39,40,41]. Immobilization ensures that the biomolecular sensing elements remain near the transducer, thereby facilitating efficient signal transduction upon analyte binding.

Moreover, enzymes are a specific class of biomolecules that are extensively used in biosensing applications due to their catalytic activity [42,43]. Enzymatic biosensors utilize enzymes as sensing elements with a transducer to generate a detectable signal proportional to the concentration of the target analyte. This resulting signal arises from enzymatic reactions that induce changes in proton concentration, gas release/uptake (e.g., ammonia or oxygen), light emission, heat release, and more [44]. The transducer converts this signal into a measurable response, like current, potential, temperature change, or light absorption, using electrochemical, thermal, or optical methods. This signal can be amplified, processed, or stored for subsequent analysis.

Additionally, antibodies are highly specific recognition elements used in immunoassays [45,46]. They can selectively bind to antigens, pathogens, toxins, or specific biomolecules, thereby forming antibody–antigen complexes. These complexes are detectable through various transduction methods, such as optical, electrochemical, or piezoelectric signals, thereby allowing for sensitive and specific detection of the target analyte.

**Figure 3:**
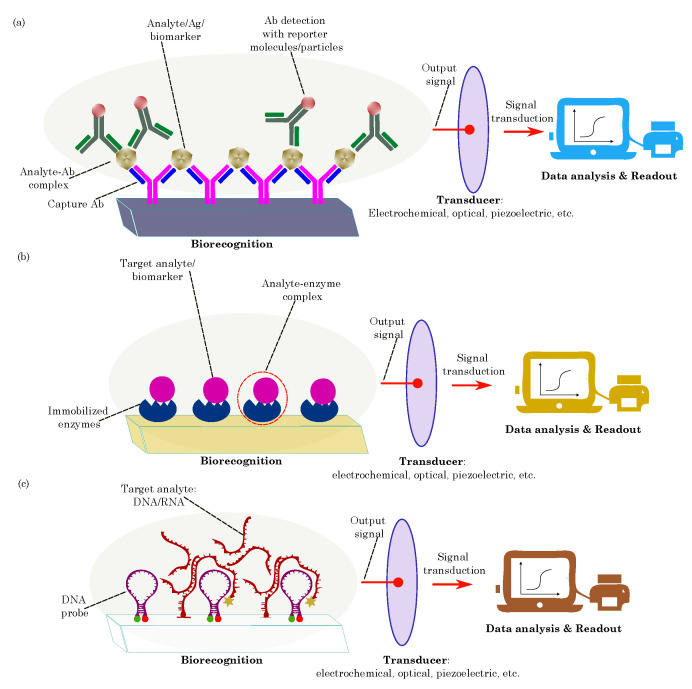
Schematics illustration of biomolecule-based biosensors: (**a**) antibody-based; (**b**) enzyme-based biosensors; (**c**) DNA/RNA-based biosensors.

Furthermore, nucleic acids, such as DNA and RNA, are utilized in nucleic acid-based biosensors [47,48,49,50]. These sensing elements recognize specific DNA sequences or RNA targets through hybridization reactions. Nucleic acid biosensors are vital for genetic analysis, disease diagnostics, and the monitoring of nucleic-acid-based biomarkers. Figure 3 illustrates the biomolecule-based biosensors.

Overall, biomolecules serve as powerful sensing elements in biosensors, thereby enabling the detection of a wide range of analytes, including proteins, nucleic acids, small molecules, and even viruses or bacteria. Their high specificity, sensitivity, and ability to function under physiological conditions make them invaluable tools in medical diagnostics, environmental monitoring, food safety, and various other applications. As research continues, integrating biomolecules into novel sensing platforms promises to revolutionize biosensing technology, thereby opening up new avenues for the precise, rapid, and cost-effective detection of analytes in diverse fields.

### 2.4. Nanomaterials

Nanomaterials are materials that are characterized by nanoscale dimensions, typically ranging from 1 to 100 nm in at least one dimension [51]. These materials exhibit unique properties that differ significantly from their bulk counterparts, thereby making them valuable for various science, engineering, and technology applications. The small size of nanomaterials results in a high surface-to-volume ratio, thus leading to enhanced reactivity and increased surface area for interactions with other materials. This unique feature allows for tailoring their physical, chemical, and mechanical properties through precise size, shape, and composition control [52].

**Figure 4:**
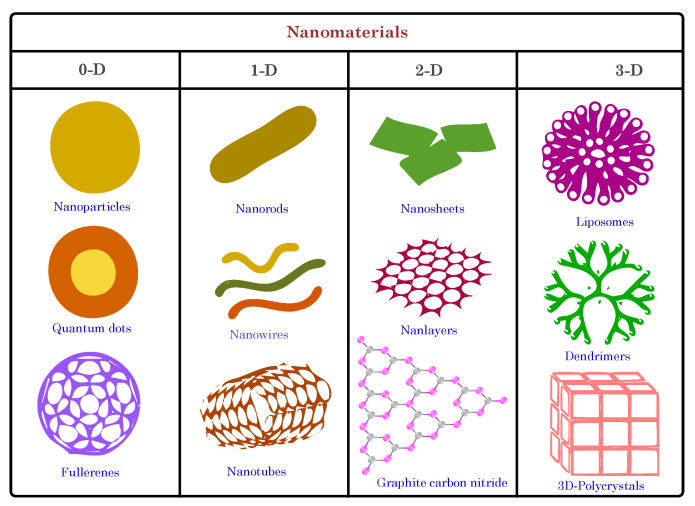
Schematic illustration of nanostructured materials classified based on dimensionality.

Based on dimensionality, nanomaterials can be categorized into four main categories: zero-dimensional (0D), one-dimensional (1D), two-dimensional (2D), and three-dimensional (3D) nanomaterials (see Figure 4). Zero-dimensional nanomaterials are nanoparticles with nanoscale dimensions in all three directions. Examples include nanoparticles and quantum dots. Nanoparticles comprise metals, metal oxides, semiconductors, polymers, and other materials. Due to their small size, nanoparticles exhibit quantum confinement effects, where their electronic and optical properties become size-dependent. This phenomenon leads to novel optical, electrical, and magnetic behaviors that are different from bulk materials. For example, gold nanoparticles exhibit unique plasmonic properties, thus making them suitable for applications in sensing and imaging [53]. Another example of 0D nanomaterials is nanocomposites, which are formed by combining nanoparticles with a matrix material to enhance specific properties. These materials integrate the unique properties of nanoparticles, such as enhanced surface area and tailored functionality, with the structural support of the matrix material [54,55]. In biosensing, nanocomposites can be engineered to create susceptible and selective sensors. Nanoparticles can act as signal amplifiers, thereby enhancing the detection signal through their distinctive optical, electrical, or catalytic properties. The matrix material provides stability, mechanical strength, and a platform for biomolecular immobilization. By judiciously selecting nanoparticle types and incorporating them into the matrix, nanocomposite-based biosensors can achieve superior sensitivity, rapid response times, and the capability to detect a wide range of analytes, including biomolecules and pathogens. For instance, incorporating hemin- and silver-coated gold nanoparticles into a graphene oxide sheet led to a highly stable catalytic nanozyme with excellent detection performance [56].

One-dimensional (1D) nanomaterials have nanoscale dimensions in two directions, while the third dimension is in the micrometer range. Carbon nanotubes (CNTs) and nanowires are noteworthy examples. CNTs are cylindrical nanostructures of carbon atoms arranged in a hexagonal lattice, thus forming a tubular shape. Due to their unique atomic arrangement, they exhibit remarkable mechanical, electrical, and thermal properties. CNTs can be single-walled (SWCNTs) or multiwalled (MWCNTs), thus exhbiting differing properties based on their structure [57,58]. SWCNTs have extraordinary electrical conductivity and can be semiconducting or metallic, thus making them ideal for various electronic and energy storage applications. MWCNTs, on the other hand, possess exceptional strength and are used in reinforcement materials. Their high aspect ratio, surface area, and tunable properties have led to their utilization in diverse fields, including nanotechnology, materials science, electronics, and biomedical applications.

Two-dimensional (2D) nanomaterials have nanoscale dimensions in one direction while the other two remain relatively larger. The most notable example is graphene, a single layer of carbon atoms arranged in a two-dimensional honeycomb lattice. Graphene has garnered immense attention for its exceptional properties and diverse applications, particularly in biosensing [59,60,61]. Its remarkable electrical conductivity, high surface area, and biocompatibility make it a promising biosensor candidate. Graphene-based biosensors can detect biomolecules with high sensitivity and specificity, as the binding of the target molecules leads to changes in their electrical properties. Its two-dimensional nature enables efficient interaction with biomolecules, thus enhancing sensor performance. Additionally, graphene’s ease of functionalization allows for the attachment of specific biomolecular recognition elements, thereby enhancing selectivity [62,63].

Three-dimensional (3D) nanomaterials are advanced structures that extend into the nanoscale in three spatial dimensions, thus offering unique properties and a high degree of control over their physical and chemical characteristics. These materials are recognized for their exceptional electroactive surface area, which allows for a higher loading capacity of recognition elements, such as antibodies or aptamers, thereby making them highly effective in targeting specific analytes, amplifying signals, and facilitating efficient biosensing with increased sensitivity and specificity. This category includes intricate hierarchical nanoscale structures and nanocomposites, which play a significant role in 3D materials [64]. A notable example of 3D nanomaterials used in biosensing is the utilization of 3D graphene nanostructures. For instance, Chen et al. [65] developed a three-dimensional electrochemical DNA biosensor utilizing silver nanoparticles decorated on a 3D graphene foam to detect CYFRA21-1 in lung cancer samples. Another study employed a graphene–metallic hybrid trimetallic nanoflower composite (3D GR/AuPtPd) to detect the epidermal growth factor receptor (EGFR) ctDNA in human serum [66]. Moreover, 3D hollow photoactive nanomaterials (such as Hollow CdS@Au nanospheres) have been instrumental in constructing multimodal biosensors for carcinoembryonic antigen detection, thereby offering increased sensitivity through enhanced light capture attributed to their unique hollow nanostructures [67].

Other types of classifications of nanomaterials (e.g., organic, carbon, and inorganic) have been extensively discussed in several published articles [68,69]. In biomedicine, nanomaterials have shown significant promise in drug delivery systems, where nanoparticles can be functionalized to carry therapeutic agents and selectively target specific cells or tissues. Additionally, nanomaterials have been utilized in diagnostic imaging and biosensing applications, where their unique properties enable the susceptible and specific detection of biological analytes. However, despite their promising advantages, nanomaterials also raise concerns regarding their potential toxicity and environmental impact [70,71,72]. Due to their small size, nanomaterials can easily penetrate biological barriers and interact with living organisms in ways that larger particles cannot. Therefore, extensive research is ongoing to understand and mitigate the potential risks of using nanomaterials.

Nanomaterials present a wealth of opportunities for groundbreaking innovations in diverse fields. Their unique size-dependent properties and versatility allow for tailoring material behavior to specific applications, thus leading to advances in electronics, medicine, energy, environmental remediation, and beyond. As nanotechnology continues to evolve, the responsible and sustainable development of nanomaterials remains critical to ensure their safe and beneficial integration into various technological and biomedical applications.

## 3. Organic Bioelectronic Devices

### 3.1. Organic Field-Effect Transistors (OFETs)

Organic field-effect transistors (OFETs) are semiconductor devices that utilize organic materials as the active channel to control the flow of charge carriers (electrons or holes) between the source and drain electrodes, and they are modulated by an externally applied electric field at the gate electrode. OFETs have gained considerable attention recently due to their potential for low-cost, flexible, and large-area electronic applications, such as displays, sensors, and integrated circuits [73,74].

**Figure 5:**
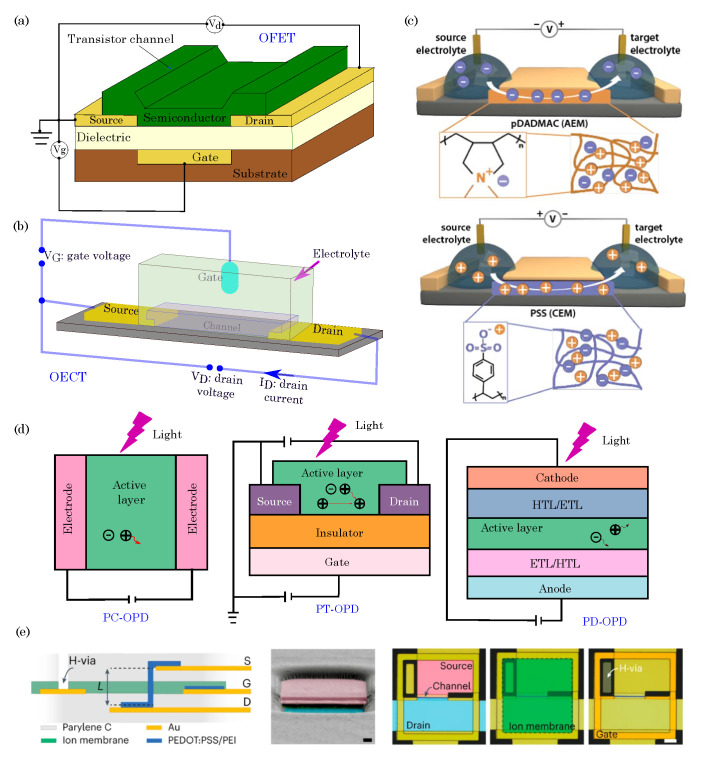
(**a**) Schematic diagram of organic field-effect transistors (OFETs). (**b**) Typical structure of an organic electrochemical transistor (OECT). Adapted from Friedlein et al. [81]. ©2018 The Authors, licensed under a Creative Commons license. (**c**) Schematic illustration of OEIP device configuration and the working principle of a potential ion-selective OEIP (**top**) and a cation-selective OEIP (**bottom**). As illustrated, applying a potential between electrodes establishes an electrochemical circuit. Within this circuit, cations or anions from a source electrolyte are selectively conveyed to a target electrolyte through an ion exchange membrane. Adapted with permission from Cherian et al. [97]. ©2019 The Authors, published by IOP Publishing Ltd. under the terms of the Creative Commons Attribution 3.0 license. (**d**) Device configurations of OPDs: organic photoconductor (PC-OPD), organic photoresistor (PT-OPD), and organic photodiode (PD-OPD). *ETL*–electron transport layer and *HTL*–hole transport layer. Adapted with permission from Liu et al. [98], ©2020 WILEY-VCH Verlag GmbH & Co. KGaA, Weinheim. (**e**) Schematic of a vIGT (left)—L: vertical channel length, S: source, G: gate, D: drain; colorized cross-section scanning electron microscopy image of a vIGT (**center**). The pink and blue regions are the source and drain contacts, and the optical micrograph displays the top view of an individual vIGT (right)—blue color: drain contact, pink: source contact, green: ion membrane. Reprinted with permission from Cea et al. [99]. ©2022 The Authors, published by Springer Nature under a Creative Commons Attribution 4.0 International License.

The basic structure of an OFET consists of three main components: the source, drain, and gate electrodes, which are all deposited on a substrate. The active channel material, typically an organic semiconductor, forms a thin film between the source and drain electrodes. A gate insulator layer separates the gate electrode from the channel material, and it is often made of an organic or inorganic dielectric [75,76]. Figure 5a illustrates the basic components of the OFET. The operation of an OFET relies on applying a gate voltage, which creates an electric field across the gate insulator and the channel material. This electric field either enhances or depletes the concentration of charge carriers in the channel, which depends on the type of OFET (n-type or p-type). In an n-type OFET, the applied gate voltage increases the concentration of electrons in the channel, while in a p-type OFET, it increases the concentration of holes. The modulation of charge carriers in the channel material leads to a change in the conductivity between the source and drain electrodes. This change in conductivity is responsible for amplifying the input signal at the gate and producing a corresponding output signal at the drain, thus making OFETs function as amplifiers or switches.

One of the significant advantages of OFETs is their compatibility with low-cost, large-area manufacturing processes, such as solution-based deposition techniques like spin coating or inkjet printing. The solution processability of organic semiconductors allows for the fabrication of flexible and stretchable devices on various substrates, including plastic and paper. The versatility of organic materials enables the ability to tailor the active channel’s electronic properties to specific application requirements. By modifying the molecular structure or introducing chemical dopants, researchers can optimize the charge transport behavior, charge carrier mobility, and overall device performance of OFETs.

OFETs find applications in various electronic devices, including electronic paper, flexible displays, RFID tags, biosensors, and logic circuits [77]. Additionally, OFET-based sensors have been developed for detecting various environmental and biological analytes, thereby making them attractive for applications in healthcare, environmental monitoring, and point-of-care diagnostics. However, despite their advantages, challenges in OFET technology remain, such as improving their charge carrier mobility, stability, and reproducibility [78,79]. Researchers continue to explore novel materials, device architectures, and fabrication techniques to enhance the performance and reliability of OFETs, thus paving the way for their integration into next-generation electronics and wearable technologies.

### 3.2. Organic Electrochemical Transistors (OECTs)

OECTs are electronic devices that utilize organic materials to enable the ion-mediated modulation of electrical conductivity. These transistors have gained significant attention due to their unique properties, such as low operating voltage, biocompatibility, and mechanical flexibility, thereby making them suitable for various applications, including biosensing, neuromorphic computing, and bioelectronics [80]. The basic structure of an OECT consists of three main components: the source, drain, and gate electrodes, which are all integrated into a substrate [81]. Figure 5b shows a typical OECT schematic diagram. The operation of an OECT relies on the electrochemical doping and dedoping of the organic channel material. When a voltage is applied to the gate electrode, ions from the electrolyte solution penetrate the organic channel material, thus creating mobile charge carriers, which are either positively charged holes or negatively charged ions. This process is known as redox doping or ion–electron coupling. The presence of mobile charge carriers in the channel material modulates its electrical conductivity, thus affecting the current flow between the source and drain electrodes [82]. The channel’s doping level can be adjusted by controlling the gate voltage, thereby amplifying the input signal and resulting in large changes in the output current [83]. This unique ion-modulated transistor behavior sets OECTs apart from traditional field-effect transistors (FETs), where the current flow is regulated by applying an electric field across the gate-insulator interface.

The OECT devices work in two modes: depletion and accumulation modes [84]. By default, the depletion mode of the OECT operates with its channel in a conducting (ON) state, thus requiring an applied gate voltage to reduce its conductivity or switch it OFF. This type of organic transistor is constructed using organic semiconductor materials (e.g., PEDOT:PSS) and relies on ion transport within the semiconductor to modulate its conductivity. In contrast, the accumulation mode of the OECT remains in a nonconducting (OFF) state until a negative gate voltage is applied. This voltage accumulates charge carriers within the organic semiconductor channel, thereby allowing the current to flow and turning the transistor ON. Like the depletion mode in OECTs, the accumulation mode in OECTs also employs organic semiconductors (e.g., p(g2T-TT)) and ion transport for its operation, but its default state is nonconductive, thus requiring a gate voltage to activate it.

One of the key advantages of OECTs is their biocompatibility, which enables their integration into biological systems without inducing significant adverse effects. This property makes OECTs ideal for interfacing with living cells and tissues, thereby enabling applications in neural interfaces and bioelectronic devices [85]. Additionally, OECTs operate at low voltages, thus reducing power consumption and enabling the development of energy-efficient electronic systems. OECTs are widely employed in biosensing applications, because they can transduce ion concentrations into electrical signals. By functionalizing the OECT channel with specific biomolecules or enzymes, the device can selectively detect and quantify target analytes, such as ions, neurotransmitters, glucose, or DNA, with high sensitivity and specificity. These biosensors find applications in medical diagnostics, environmental monitoring, and wearable health monitoring. Furthermore, OECTs have been utilized in neuromorphic computing, where they mimic the behavior of biological neurons in artificial neural networks. Their ion-mediated operation allows for dynamic signal processing and synaptic-like behavior, thus making them promising candidates for brain-inspired computing and pattern recognition tasks.

While OECTs offer numerous advantages, challenges remain in optimizing their stability, reproducibility, and scalability for large-scale production. Researchers continue to explore novel materials, device architectures, and fabrication methods to enhance the performance and reliability of OECTs, thus paving the way for their widespread adoption in cutting-edge electronic and bioelectronic technologies.

### 3.3. Organic Electronic Ion Pumps (OEIPs)

Organic electronic ion pumps represent a burgeoning area of research in bioelectronics, where the principles of organic materials and electronics converge to create advanced systems for ion transport [86,87]. These ion pumps utilize organic materials with specific ion-selective properties to enable the controlled and precise transport of ions, such as cations, anions, protons, or other charged species. The underlying principle involves utilizing organic materials that can change their state, conductivity, or permeability when exposed to external stimuli such as voltage or chemical signals. By applying an electrical potential, these materials can effectively regulate the movement of ions across a membrane or interface. Figure 5c depicts the typical device configuration of a potential ion-selective OEIP and a cation-selective OEIP. As shown, an OEIP comprises two electrodes separated by an ion-exchange membrane. When a voltage is applied between the two electrodes, one of which is positioned beneath the ion reservoir and the other situated at the target area, cations (or anions) migrate from the reservoir through the respective exchange membrane to the delivery site [88].

The OEIPs’ capability to manipulate ion transport holds significant implications across diverse domains, ranging from addressing therapeutic challenges through targeted drug delivery and neural modulation to applications in biotechnology and bioengineering. Examples of OEIPs encompass triggering cell signaling in vitro [89,90], controlling epileptiform activity in brain slice models [91], influencing sensory functions in vivo [92], serving as pain therapy in awake animals [93], and even regulating plant growth through the delivery of phytohormones [94].

Organic electronic ion pumps offer several advantages, including biocompatibility, flexibility, and the potential for miniaturization. These properties make them well suited for integration into bioelectronic devices and implantable systems [95,96]. OEIPs can be designed to work in tandem with other components like sensors, actuators, and communication modules. This integration allows for dynamic feedback loops, thus enabling real-time adjustments in ion transport based on physiological responses or external triggers. As research advances, the development of organic electronic ion pumps has the potential to revolutionize the field of bioelectronics, thus opening up new avenues for creating smart and responsive biointegrated systems that interface seamlessly with biological environments and hold promise for a range of medical, therapeutic, and biotechnological applications.

### 3.4. Organic Photodetectors (OPDs)

Organic photodetectors (OPDs) are optoelectronic devices that convert incident light into electrical signals through the photoelectric effect, thus utilizing organic materials as the active absorbing layer. These devices have gained significant attention due to their potential for low-cost, flexible, and large-area optoelectronic applications, including image sensors, photodiodes, and light detectors [100].

The basic structure of an organic photodetector typically comprises an organic semiconductor layer sandwiched between two electrodes, which acts as the anode and cathode. The organic semiconductor layer absorbs photons from incident light, thereby generating electron–hole pairs in the material. Depending on the type of OPD, either electrons or holes are transported through the organic layer to the respective electrodes. The operation of an OPD is based on the efficient generation, separation, and collection of photoexcited charge carriers. When photons with energy equal to or greater than the semiconductor band gap are absorbed, excitons (electron–hole pairs) are created. These excitons must be efficiently dissociated into free-charge carriers to generate a photocurrent. To enhance exciton dissociation, OPDs often incorporate donor–acceptor heterojunctions, where the energy levels of the donor and acceptor materials promote efficient charge separation. The photocurrent generated in the organic layer is collected at the electrodes, and the magnitude of the photocurrent is proportional to the intensity of the incident light. Figure 5d shows the three different architectures of the OPDs, namely, photoconductor-based OPDs (PC-OPDs), phototransistor-based OPDs (PT-OPDs), and photodiode-based OPDs (PD-OPDs). The PT-OPDs comprise three electrodes: the gate, source, and drain. In contrast, the PC-OPDs and PD-OPDs are configured based on two electrodes (i.e., an anode and a cathode). PC-OPDs leverage photoconductivity in organic materials to detect light, thus offering sensitivity across a wide spectrum. PT-OPDs employ a transistor structure for amplified sensitivity, thereby making them ideal for low-light conditions. PD-OPDs combine organic semiconductors with photodiode principles, thus delivering high-speed and efficient light detection, which is crucial for applications like optical communication and rapid imaging. Each OPD type caters to specific needs, thus providing a versatile toolkit for various optoelectronic applications.

Apart from sandwich types, planar-type photodetectors have also been used. These photodetectors are semiconductor devices with a planar structure designed for efficient light detection and conversion into electrical signals. These devices, typically made from semiconductor materials like silicon (Si) and gallium arsenide (GaAs) [101,102,103], operate on a fundamental principle where incident photons with energy greater than the semiconductor’s band gap generate electron–hole pairs when they strike the device’s surface. The resulting free electrons and holes are then separated and collected by an internal electric field, thus creating a photocurrent or a change in voltage, which is directly proportional to the intensity of the incident light. Planar-type photodetectors encompass various designs, including photodiodes, phototransistors, and avalanche photodiodes [104,105,106]. Photodiodes collect the separated carriers directly, thus offering a linear response to incident light. Phototransistors amplify the signal by using the generated carriers to control a larger current flow, while avalanche photodiodes, intended for applications requiring high sensitivity, leverage avalanche multiplication to produce a substantial number of charge carriers. These photodetectors are extensively applied in optical communication systems, imaging devices, optical sensors, and many applications demanding light detection.

OPDs exhibit high responsivity, a low dark current, and fast response times, thus making them suitable for a wide range of light detection applications. One of the key advantages of OPDs is their compatibility with solution-based deposition techniques, thereby enabling the fabrication of large-area and flexible devices on various substrates. The tunability of organic materials allows for optimizing their light absorption properties to match specific wavelengths or spectral ranges, thus making OPDs versatile for various optical sensing and imaging applications. OPDs are used in diverse optoelectronic devices, such as image sensors [107,108,109], light-sensitive arrays, photodetector arrays [110], and position-sensitive detectors [111,112,113]. They find applications in digital cameras, medical imaging, light-based communication systems, and optical sensors for environmental monitoring and industrial applications. Additionally, the organic materials’ flexibility and lightweight nature enable the development of wearable and conformable photodetectors for wearable health monitoring, biometric sensing, and smart textiles.

Despite their advantages, challenges in OPD technology include improving the external quantum efficiency, enhancing the stability of organic materials under prolonged light exposure, and achieving high-speed response times for rapid optical sensing applications [98]. Researchers are actively exploring novel organic materials, device architectures, and engineering strategies to overcome these challenges and unlock the full potential of organic photodetectors in the emerging field of organic optoelectronics.

### 3.5. Organic Bioelectronic Implants

Organic bioelectronic implants are advanced medical devices integrating organic electronic materials and components into living tissues to enable various therapeutic or diagnostic functionalities. These implants represent a cutting-edge field of research and development in the intersection of organic electronics and biomedicine, thereby offering unique advantages for medical applications [114,115].

Organic bioelectronic implants constitute a complex assembly of crucial components aimed at interfacing with biological systems while delivering therapeutic or monitoring functions. Central to their design are organic semiconductors, conductive polymers like poly(3,4-ethylene dioxythiophene): poly(styrene sulfonate) (PEDOT:PSS), and specialized organic electronic materials meticulously chosen for their biocompatibility, mechanical flexibility, and ability to seamlessly integrate with biological tissues, all while evading significant immune responses [93]. These materials serve as the foundation for the implant’s active elements.

Organic bioelectronic implants exhibit adaptability by incorporating sensors to monitor vital physiological parameters like pH, temperature, glucose levels, or specific biomarkers. Additionally, they integrate stimulating components such as electrodes or transducers that are capable of delivering targeted electrical or chemical signals. These signals serve therapeutic objectives such as deep brain stimulation or promoting neural regeneration. To ensure the longevity and efficacy of the implant, it is encapsulated within biocompatible materials or coatings. This encapsulation acts as a protective barrier against unwanted interactions with the surrounding biological environment. In a recent example, Cea et al. [99] developed a tiny, fully organic bioelectronic device that acquires and transmits brain signals and is self-powered. The device is about 100 times smaller than a human hair and is based on an IGT (internal-ion-gated organic electrochemical transistor) architecture, as well as the vIGT (vertical-internal-ion-gated organic electrochemical transistor) architecture that incorporates a vertical channel made of PEDOT:PSS and a miniaturized water conduit (H-via) from the surface of the device through the ion membrane layer to permit channel hydration, thereby demonstrating long-term stability, high electrical performance, and low-voltage operation to prevent biological tissue damage. Figure 5e demonstrates the device architecture schematics, SEM image, and optical micrograph of the vIGT.

Furthermore, these implants harness wireless communication, thereby enabling connectivity with external devices for data collection, remote control, and programming. This breakthrough promises a revolution in patient monitoring and treatment optimization, as demonstrated by recent studies [116,117,118]. They also employ innovative power management systems, including energy harvesting and wireless charging, thus ensuring sustainable operation and reducing the need for frequent battery replacements. An additional advantage lies in the mechanical flexibility of organic bioelectronic implants, which enable seamless integration with irregular and dynamic tissue shapes and movements. This adaptability proves invaluable when implants are placed in soft, curved body regions like the brain, heart, or spinal cord.

Moreover, recent advancements have led to the development of biodegradable organic bioelectronic implants. These designs gradually dissolve over time, thus minimizing harm to surrounding tissues and eliminating the need for additional surgical removal. These implantable bioelectronic devices offer immense potential across diverse medical applications. Organic sensors can precisely monitor drug release rates and tailor dosages for personalized drug administration. Moreover, they facilitate tissue regeneration by offering electrical or biochemical cues to spur cell growth and tissue repair. Notably, these devices find application in neuroprosthetics, including cochlear implants for hearing restoration and retinal implants for vision enhancement [119]. Additionally, they are employed for simulating peripheral nerves to treat disorders that are resistant to traditional pharmacological interventions.

As organic bioelectronic implants advance, ongoing research focuses on optimizing their biocompatibility, stability, and long-term functionality, as well as addressing challenges related to immune responses, long-term biointegration, and regulatory approvals. With continued innovations, organic bioelectronic implants have the potential to revolutionize personalized medicine, thus ushering in a new era of advanced healthcare and improved quality of life for patients.

## 4. Fabrication Methods

Organic bioelectronic devices are predominantly fabricated/patterned using several approaches, such as organic thin-film deposition methods, patterning techniques, 3D printing, and organic synthesis.

**Figure 6:**
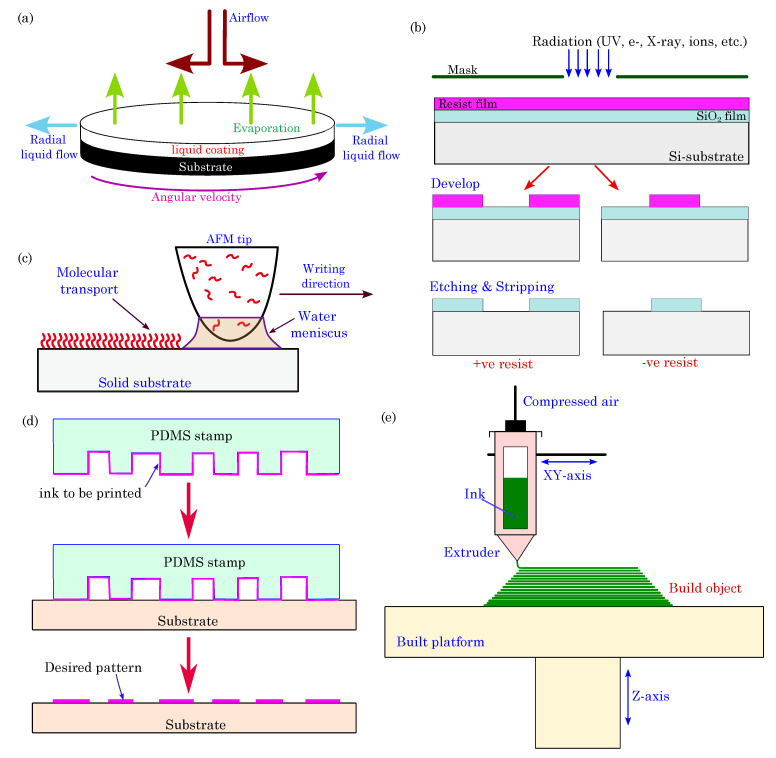
Schematic diagram of various fabrication methods: (**a**) spin-coating process; (**b**) photolithography; (**c**) dip-pen nanolithography (DPN); (**d**) microcontact printing (μCP); (**e**) direct ink writing (DIW).

Organic thin-film deposition: These methods are widely used for depositing thin films of organic materials on substrates with controlled thickness and uniformity. One common technique is spin coating. In spin coating, an organic material solution, such as semiconductors, conductive polymers, or other active components, is deposited onto a flat substrate, typically a silicon wafer or glass, which can be further integrated into a device (see schematics in Figure 6a). As the substrate spins at high speeds, centrifugal forces evenly distribute the material to result in a thin, uniform film. The spin coating offers precise control over the thickness and quality of the deposited organic films, thereby allowing researchers to optimize these devices’ electrical and optical properties. This method is ideal for producing the organic semiconductor layers used in devices like organic field-effect transistors (OFETs) and organic photodetectors [120,121]. With its scalability, cost-effectiveness, and ongoing refinements, spin coating plays a central role in various applications, from flexible electronics to medical diagnostics and wearable health monitoring, thereby ensuring the advancement of organic bioelectronics in diverse fields.

Vacuum evaporation is another thin-film deposition method. It facilitates the precise deposition of organic materials onto various substrates under reduced pressure conditions. In this process, organic materials, such as semiconductors, conductive polymers, and other key bioelectronic components, are heated to their vaporization points and then allowed to condense onto the target substrate, thereby creating thin organic films with exceptional uniformity and precise thickness control. This level of control is indispensable in developing organic electronic devices, including organic field-effect transistors (OFETs) and organic photodetectors, where the properties of the organic layer directly influence the device performance. Vacuum evaporation enables the sequential deposition of multiple organic layers, thereby making it possible to design complex device architectures. This capability is invaluable, as organic bioelectronic devices often require distinct functional layers for sensing, signal processing, and data transmission. Additionally, vacuum evaporation is a low-temperature deposition technique that safeguards the structural integrity of heat-sensitive organic materials. It also provides a pristine vacuum environment that minimizes contamination, thus ensuring the quality of the deposited organic films. In the realm of organic bioelectronics, vacuum evaporation plays a critical role in manufacturing devices like biosensors, organic photovoltaics, and implantable bioelectronic systems. For instance, vacuum evaporation is often used in OLED fabrication [122,123].

Patterning techniques: Several methods are employed for patterning electrodes for organic electronic devices, such as photolithography, e-beam lithography, dip-pen lithography, inkjet printing, microcontact printing, screen printing, direct ink writing, laser writing, etc.

*Photolithography*: It is a well-established technique for patterning organic materials at micron and submicron scales. The photolithography process begins with a substrate, typically made of silicon or glass, that is coated with a layer of photoresist, which is a photosensitive organic material. A photomask containing the desired pattern is placed near the photoresist-coated substrate, and the entire assembly is exposed to ultraviolet (UV) light. The exposed regions undergo a chemical change, thereby making them either more soluble (in the case of positive photoresists) or less soluble (for negative photoresists) in a developer solution, which depends on the type of photoresist used. The developer solution is applied to the substrate, thus removing the selected areas and leaving behind the desired pattern (see schematics in Figure 6b). Photolithography stands out due to its exceptional resolution and accuracy, thereby making it capable of crafting intricate micro- and nanoscale structures relevant to organic bioelectronics. The adaptability of this technique to a range of organic materials facilitates the fabrication of diverse bioelectronic components. Nonetheless, the cautious handling of organic materials is essential, as some may be sensitive to UV exposure and chemical developers. Additionally, the meticulous design of photomasks is imperative to achieve the desired patterns. Photolithography is employed in organic bioelectronic device fabrication to create features like electrodes, sensor structures, and microfluidic channels [124,125,126,127].

*Electron-Beam (e-beam) Lithography*: Electron-beam lithography or EBL is an advanced nanofabrication technique that operates on the fundamental principle of using a focused beam of electrons to create incredibly fine patterns and structures at the nanometer scale. It has found applications in various fields, including semiconductor device fabrication, nanotechnology, and microelectromechanical systems (MEMS). Unlike conventional photolithography, EBL can achieve unparalleled resolution by crafting features with dimensions down to just a few nanometers. This capability arises from its direct writing process, where a precisely controlled electron beam moves across an electron-sensitive resist on a substrate to define intricate custom patterns. While slower and more complex than some alternatives, e-beam lithography is crucial in developing advanced nanoscale devices, specialized structures in research laboratories, and creating masks and photomasks for semiconductor manufacturing.

*Dip-Pen Nanolithography (DPN)*: DPN is an advanced nanofabrication technique that leverages the precision of scanning probe microscopy (SPM) for the controlled deposition of molecules, nanoparticles, or biomolecules onto a substrate with nanometer-scale precision. In this method, an atomic force microscope (AFM) tip coated with an “ink” material is submerged, or “dipped”, into the ink and then brought into contact with a substrate under the guidance of the AFM (schematics in Figure 6c). DPN is renowned for its extraordinary sub-10 nm resolution, thereby making it a pivotal tool in various domains, such as nanoelectronics, nanophotonics, and nanobiotechnology. Its remarkable versatility extends to the patterning of diverse materials, including conducting polymers, biological compounds like proteins or DNA, nanoparticles, and more, thus enabling the creation of various structures, from lines and dots to intricate two-dimensional and three-dimensional designs. DPN finds applications across several domains: in nanoelectronics for the development of nanoscale electronic components and features on semiconductor chips; in nanophotonics for crafting optical devices, photonic circuits, and metamaterials; in biosensing for creating highly sensitive and specific biosensors; in surface functionalization for engineering specific surface properties; and in nanomaterial synthesis for the precise control of nanoparticle properties. While DPN offers exceptional precision, it can be a relatively slow and serial process, thus limiting its application for large-scale manufacturing, and the choice of ink, tip, substrate, and environmental conditions significantly influence the pattern quality and reproducibility.

*Microcontact Printing* (μCP): μCP is a widely used soft lithography technique employed for precise and controlled deposition of materials, often in the form of self-assembled monolayers (SAMs), on a substrate. The process is akin to conventional rubber stamp printing but on a micro- and nanoscale. μCP employs an elastomeric stamp, usually made of polydimethylsiloxane (PDMS), that is engineered with relief microstructures or patterns on its surface. The stamp is coated with an “ink” or material, which adheres only to the relief patterns. The stamp is then gently brought into contact with a substrate, thereby transferring the material onto the substrate in the desired pattern (see schematics in Figure 6d). This process offers several advantages, including simplicity, cost-effectiveness, and the ability to create well-defined and precisely placed chemical patterns on various substrates, including metals, semiconductors, and organic materials. μCP is particularly valuable for creating surface chemistry modifications and developing biomolecule arrays, as well as microscale patterning for various applications, including biosensors, microelectronics, and microfluidics. However, μCP also has some limitations. It may be less suitable for large-scale or high-throughput manufacturing processes, as it is inherently a serial process. The resolution of μCP depends on the stamp’s relief structures and may not achieve the sub-10-nanometer scale of some advanced lithography techniques. Additionally, controlling the uniformity of the ink layer and ensuring consistent contact between the stamp and substrate can be challenging. Despite these limitations, microcontact printing remains a powerful tool for many micro- and nanofabrication applications, particularly in research and prototyping scenarios.

*Inkjet Printing*: Inkjet printing, a highly versatile technique, has become integral to the realm of organic bioelectronics. The process involves depositing minuscule ink droplets onto a substrate, thus enabling controlled patterning of various functional materials, including organic semiconductors, conductive polymers, and biologically relevant molecules. Its prominence in this field stems from multiple advantages, such as exceptional precision and resolution, broad material compatibility, reduced material wastage due to its additive nature, high levels of customization to adapt complex designs for specific applications, noncontact printing, and scalability that accommodates everything from research-level prototyping to large-scale production [128,129,130,131]. Inkjet printing plays a pivotal role in fabricating components for organic bioelectronic devices, including sensors, transistors, and electrochemical systems, and it excels in the precise deposition of biomolecules crucial for biosensing and detection applications. This technology is a cornerstone in developing advanced medical diagnostics, wearable health monitoring devices, and implantable bioelectronics, thereby promising significant contributions to healthcare and environmental monitoring.

*Laser Writing*: Laser writing, also known as laser-induced forward transfer (LIFT), is an advanced microfabrication technique that employs a high-intensity laser beam to transfer material from a donor layer to a receiver substrate, thereby enabling the precise deposition of micro- or nanoscale features. A laser pulse generates a shockwave within the donor material, thus propelling a small amount of material toward a transparent receiver substrate placed above it. This method offers exceptional precision, thereby allowing for fine control over the position and size of the deposited material and making it ideal for creating intricate patterns, microarrays, and electronic devices. One of its significant advantages is versatility, as it can be used with various materials, including organic polymers, conductive substances, and biological compounds, thereby making it suitable for applications ranging from organic electronics to biosensors. Due to its non−contact nature and direct writing capabilities, laser writing is precious for handling sensitive materials and enabling rapid prototyping. With the potential to achieve submicron resolutions, this technique has widespread applications in microelectronics, flexible electronics, organic photovoltaics, microfluidics, and tissue engineering, where high-resolution and customized structures are paramount for research, development, and specialized manufacturing processes.

3D Printing: The field of bioelectronics has witnessed remarkable progress with the integration of 3D printing technologies. These technologies are known for their streamlined processes, which empower the creation of intricate three-dimensional structures with exceptional precision, scalability, and adaptability [132,133,134]. Various 3D printing techniques, including fuse deposition modeling (FDM), stereolithography (SLA), digital light processing (DLP), selective laser sintering (SLS), and direct ink writing (DIW), have been instrumental in patterning and fabricating materials with diverse strategies. Nevertheless, many of these technologies are often associated with specific material classes, such as thermoplastic polymers for FDM, photopolymer resins for SLA and DLP, and powdered polymers or metals for SLS, which impose limitations on the customization of the inks. Within this landscape, DIW, an extrusion-based 3D printing technique that constructs 3D structures layer-by-layer through the precise deposition of inks via fine nozzles (schematics in Figure 6e), has emerged as the most versatile 3D printing technology, thus offering unprecedented capabilities for the development of bioelectronics. These inks may encompass various materials, spanning metals, ceramics, polymers, carbons, and even biocompatible substances such as cells or gels. The DIW printer follows a computer-generated design to create intricate and customized objects layer by layer [135].

Chemical Methods: Organic bioelectronic devices can also be fabricated through diverse chemical methods, including polymerization, chemical vapor deposition (CVD), and self-assembly [136,137,138,139,140,141,142]. These methods allow for precise control over the molecular structure of the materials, thereby enabling the design of custom organic semiconductors, conductive polymers, and biocompatible coatings. Polymerization involves the creation of organic materials through the reaction of monomers, thus resulting in polymers with desired properties. CVD entails depositing thin films of organic materials from vapor-phase precursors, thus ensuring uniform and controlled material growth. Self-assembly allows organic molecules to spontaneously arrange into ordered structures, which can be finetuned for targeted functionalities [143,144].

Table 1 summarizes the fabrication techniques used for fabricating organic bioelectronic devices. These fabrication methods provide versatility when designing organic bioelectronic materials with unique characteristics, such as high sensitivity, flexibility, and biocompatibility. By leveraging organic thin-film deposition and organic synthesis techniques, researchers can engineer materials tailored to the requirements of biosensing, medical diagnostics, and wearable health monitoring applications, among others. Continued advancements in organic bioelectronic material fabrication hold great potential to revolutionize the landscape of bioelectronics and contribute to breakthroughs in medical technologies and personalized healthcare.

## 5. Biosensing Mechanisms

**Figure 7:**
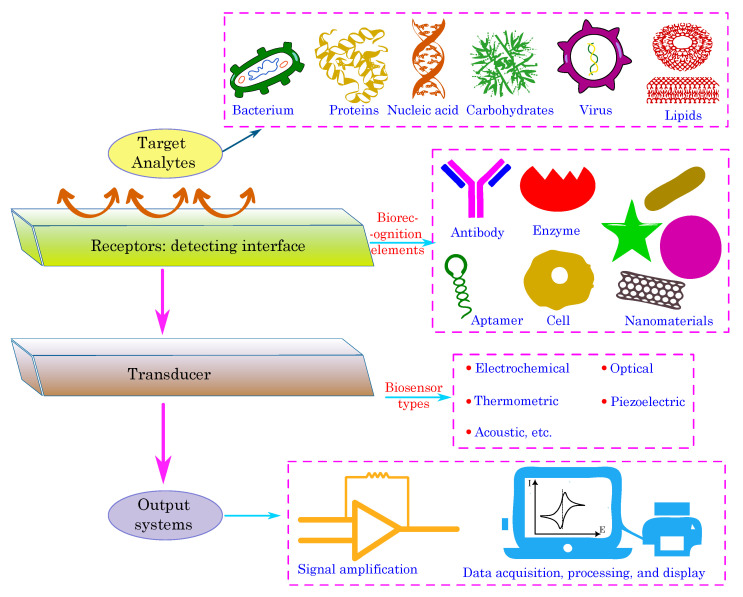
Schematic illustration of key components of a typical biosensor.

A typical biosensor comprises several fundamental components: the target analytes, receptors or biorecognition elements, a transducer, and output systems [175,176]. The target analyte is the specific substance under investigation, such as glucose, ammonia, alcohol, or lactose. Bioreceptors are biomolecules or biological entities that are capable of recognizing and binding to the target analyte. Examples of biorecognition components include enzymes, cells, aptamers, DNA/RNA strands, and antibodies. The role of the transducer is to convert the biorecognition event into a measurable signal, typically in the form of an electrical signal, which correlates with the quantity or presence of the chemical or biological target. This conversion process is known as signalization. Transducers generate optical or electrical signals that directly correspond to the interactions between the analytes and bioreceptors. Finally, output systems encompass signal processing, amplification, and display units, thereby facilitating the interpretation and presentation of the biosensor’s results. Figure 7 illustrates the components of the typical biosensor.

### 5.1. Electrochemical Sensing

Electrochemical sensing is a powerful mechanism that is utilized in organic bioelectronics for detecting and quantifying various biomolecules and chemical species. This sensing platform measures electrical signals generated during electrochemical reactions at the interface between the organic material and the target analyte. Organic electrochemical sensors offer high sensitivity, rapid response times, and excellent selectivity, thereby making them valuable medical diagnostics, environmental monitoring, and point-of-care testing tools. The fundamental principle behind electrochemical sensing in organic bioelectronics lies in the redox properties of organic materials, which can undergo reversible electron transfer reactions [177,178]. These redox-active organic materials, such as conducting polymers, redox enzymes, or organic nanoparticles, are integrated into the sensing platform to act as the transducer element. Electrochemical sensing involves two main components: an electrode and a redox reaction with the target analyte. The sensing platform typically comprises working (or indicator electrode), reference, and counter electrodes (in some cases, the two-electrode system can be used for electrochemical sensing) [179,180]. The working electrode (WE) is coated with the redox-active organic material, where the electrochemical reaction with the target analyte occurs. The reference electrode (RE) maintains a constant potential against which the working electrode’s potential is measured. The counter electrode (CE) completes the electrical circuit and balances the current flow during the electrochemical reaction. When the target analyte comes into contact with the redox-active organic material on the working electrode, it induces an electrochemical reaction. The redox-active organic material is reversibly oxidized or reduced, thus transferring electrons between the analyte and the electrode surface. This electron transfer generates an electrical signal, such as a current or potential, which is proportional to the concentration of the target analyte. Different electrochemical sensing modalities employed in organic bioelectronics include the following.

**Figure 8:**
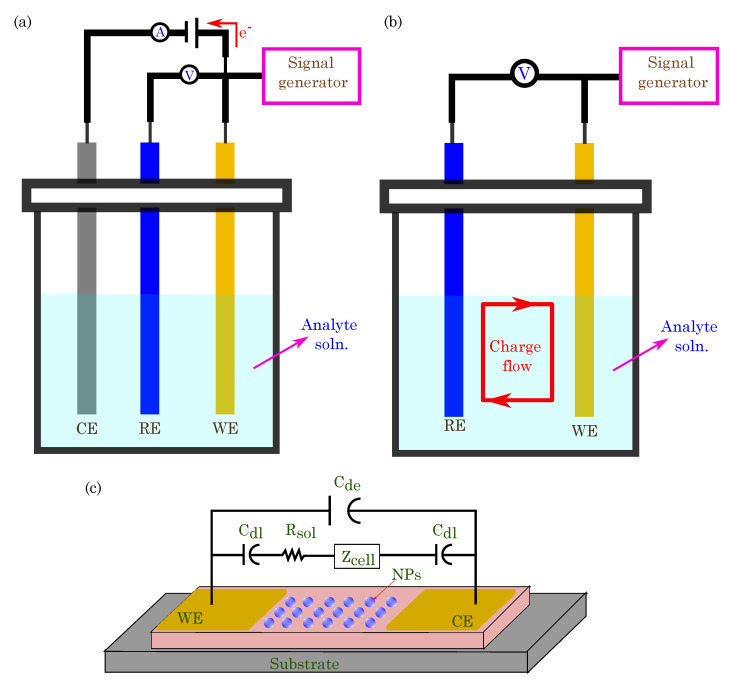
SchematicsS configuration of different types of electrochemical sensors: (**a**) amperometric/voltammetric biosensor, (**b**) potentiometric biosensor, (**c**) impedimetric biosensor (Cdl is the double-layer capacitance of the electrodes, Rsol is the resistance of the solution, Cde is the capacitance of the electrode, Zcell is the impedance introduced by the bound nanoparticles). Adapted from Naresh and Lee [176], ©2021 MDPI.

Amperometric Sensing: Amperometric biosensors are a type of electrochemical biosensor used for quantitatively detecting and analyzing biological analytes. These biosensors rely on the measurement of the current generated from an electrochemical redox reaction at the sensor’s working electrode surface when the target analyte interacts with a biorecognition element (such as enzymes, antibodies, or nucleic acids) that is immobilized on the electrode. The basic setup of an amperometric biosensor typically consists of three main components: the working electrode, the reference electrode, and the counter electrode. The biorecognition element is immobilized in the working electrode, and the redox reaction occurs upon the binding of the target analyte. The reference electrode maintains a constant potential, while the counter electrode completes the electrical circuit, thus allowing the flow of electrons during the redox reaction. When the target analyte binds to the biorecognition element on the working electrode’s surface, it triggers the redox reaction, thus producing or consuming electroactive species (e.g., hydrogen peroxide or oxygen). The current generated from this redox reaction is directly proportional to the concentration of the target analyte in the sample. As the concentration of the analyte changes, the current also varies, thus providing quantitative information about the analyte concentration. Figure 8a shows the schematics of amperometric-based biosensors.

Voltammetric Biosensing: Voltammetric biosensors are a type of electrochemical biosensor that rely on the measurement of the current as a function of an applied voltage or potential at the sensor’s working electrode. These biosensors use the principles of voltammetry to detect and quantify the target analyte in a sample. The basic setup of a voltammetric biosensor includes a working electrode coated with a biorecognition element, a reference electrode, and a counter electrode. When an increasing or decreasing voltage is applied to the working electrode, a redox reaction occurs at the electrode surface, involving the oxidation and reduction of electroactive species. In the presence of the target analyte, the biorecognition element at the working electrode surface interacts with the analyte, thereby leading to changes in the redox reaction of the electroactive species. These changes result in variations in the current measured at the working electrode, which can be correlated with the concentration of the target analyte in the sample.

Potentiometric Sensing: Potentiometric biosensors are a type of electrochemical biosensor used for the quantitative detection and analysis of biological analytes. Unlike amperometric biosensors that measure the current generated from a redox reaction, potentiometric biosensors rely on measuring potential or voltage changes at the sensor’s working electrode surface when the target analyte interacts with a biorecognition element. The basic setup of a potentiometric biosensor includes a working electrode and a reference electrode (Figure 8b) [181]. The working electrode is coated with biorecognition elements, such as enzymes, antibodies, or nucleic acids, which interact with the target analyte in the sample. The reference electrode maintains a constant potential, thus serving as a reference point to measure the potential changes at the working electrode. When the target analyte binds to the biorecognition element on the working electrode’s surface, it changes the local charge distribution, thus resulting in a potential difference. This potential change is directly related to the concentration of the target analyte in the sample. Potentiometric biosensors offer several advantages, including high specificity, label-free detection, and simple instrumentation [182]. They are particularly suitable for measuring ion concentrations, pH levels, and other analytes directly affecting local charge distribution.

Impedimetric Sensing: Impedimetric biosensors are a type of electrochemical biosensor that measure the electrical impedance or resistance changes at the sensor’s working electrode surface in response to the interaction between a biorecognition element and the target analyte (Figure 8c). This label-free and real-time detection method is highly sensitive and enables the study of various biomolecular interactions, thereby making it valuable in biosensing applications. The basic setup of an impedimetric biosensor includes a working electrode that is coated (or functionalized) with a biorecognition element (such as antibodies, enzymes, or DNA probes), a reference electrode, and a counter electrode. When the target analyte (e.g., antigen, enzyme substrate, or complementary DNA strand) binds to the biomolecules, it causes a change in the dielectric properties or the electrical double layer at the electrode surface. When an AC signal is applied to the working electrode, the impedance of the sensor changes due to the binding events between the biorecognition element and the target analyte. These changes in impedance are then measured and correlated with the concentration of the target analyte in the sample.

Impedance-based biosensors can be classified into two main types: capacitive and conductive. Capacitive impedance biosensors rely on changes in the dielectric properties of the interface between the sensing element and the target analyte. When the analyte binds to the immobilized biomolecules, it alters the dielectric constant and thickness of the insulating layer, thereby leading to changes in the electrode’s capacitance. These changes are then measured and related to the concentration of the analyte. Conductive impedance biosensors work based on changes in the electrical resistance at the electrode interface. The binding of the analyte to the sensing element causes changes in the electrical properties of the surface layer, thus leading to variations in resistance. These changes are measured to quantify the analyte concentration.

Impedimetric biosensors offer several advantages, including label-free detection, high sensitivity, and real-time monitoring capabilities. They are particularly suitable for detecting biomolecular interactions, such as antigen–antibody binding, enzyme–substrate reactions, and nucleic acid hybridization. Impedimetric biosensors are versatile and can detect various analytes, including proteins, nucleic acids, and small molecules. Although highly sensitive, impedance-based biosensors may encounter challenges related to specificity, as they could exhibit crossreactivity with similar molecules. Careful calibration is essential due to the influence of surface effects on impedance measurements. Additionally, complex sample matrices, such as blood or soil, might interfere with impedance measurements, thus potentially impacting result accuracy. Addressing these issues is crucial to ensure the reliability and applicability of impedance-based biosensors in various scientific and biomedical applications.

### 5.2. Optical Sensing

Optical sensing utilizes the interaction between light and organic materials to detect and quantify biological or chemical analytes. These sensing platforms employ organic materials, such as organic semiconductors, fluorescent dyes, or organic nanoparticles, which are integrated into photonic or optoelectronic devices to facilitate the sensitive and selective detection of target molecules. The fundamental principle behind optical sensing in organic bioelectronics relies on the optical properties of the organic materials, which can absorb, emit, or scatter light in response to changes in their environment. Within the realm of optical biosensors, various types have been developed, with each catering to specific applications and detection requirements [183,184].

**Figure 9:**
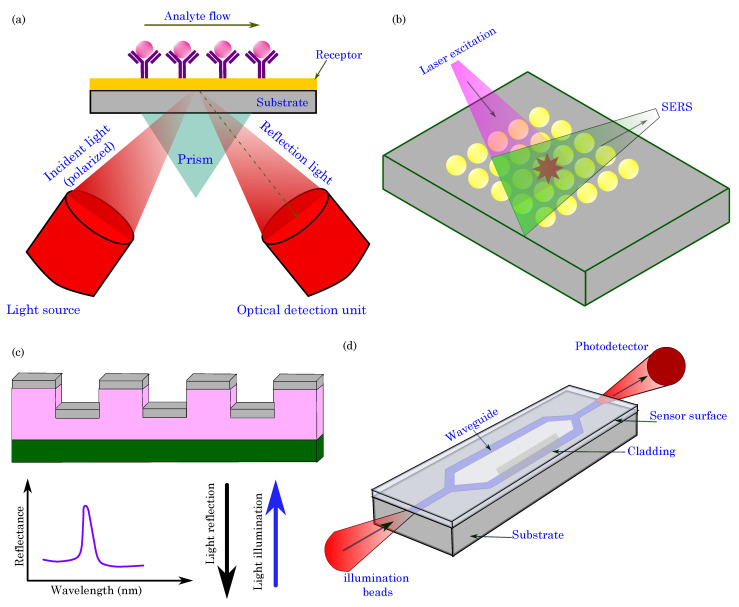
Schematics diagrams of optical biosensors. (**a**) Surface plasmon resonance (SPR) biosensor. (**b**) Surface-enhanced Raman scattering (SERS) biosensor. (**c**) Illustration of the sensing mechanism of a photonic crystal (PC) biosensor. Adapted from Chen et al. [190], ©2020 MDPI. (**d**) Optical waveguide (Mach–Zehnder) interferometer biosensor, adapted with permission from Kozma et al. [191], ©2014 Elsevier B.V.

Surface plasmon resonance (SPR) biosensors, one of the most well-known optical biosensors, rely on the principle of plasmon resonance, which occurs when light interacts with the collective oscillations of electrons on a metal surface [185]. Changes in the refractive index due to binding events on the sensor surface lead to alterations in the resonance angle, thereby enabling the label-free and real-time detection of molecular interactions. Figure 9a shows the schematic of the SPR-based biosensor. SPR biosensors find applications in drug discovery, medical diagnostics, and environmental monitoring [186].

Surface-enhanced Raman scattering (SERS) biosensors leverage the enhancement of Raman scattering signals when molecules are adsorbed on roughened metal surfaces. Molecules adsorbed on these surfaces generate unique Raman spectra, thereby enabling molecular identification and quantification (Figure 9b). SERS stands out as an exceptionally sensitive method for identifying low-concentration molecules. It excels in detecting various substances, such as DNA, microRNA, proteins, blood components, and bacteria. Furthermore, it facilitates the detection and characterization of individual cells, aids in bioimaging, and plays a pivotal role in diagnosing various diseases. Its unique ability to offer extensive structural insights into biological analytes adds significant value to the field of analytical science and diagnostics [187].

Fluorescence is a widely used optical phenomenon for biosensing [188]. In fluorescence-based optical sensing, organic fluorescent dyes or fluorophores are used as the sensing elements. When excited with a specific wavelength of light, these fluorescent molecules absorb energy and become excited to higher energy states. Subsequently, they release this excess energy through fluorescence emission at a longer wavelength. The intensity of the emitted fluorescence signal is directly proportional to the concentration of the target analyte, thereby enabling quantitative detection. Fluorescence-based organic bioelectronic sensors offer high sensitivity and excellent selectivity, thus making them valuable tools in molecular imaging, cellular assays, DNA sequencing, protein–protein interaction studies, and diagnostic applications.

Photonic crystal optical biosensors harness the unique properties of photonic crystals to enable the sensitive and specific detection of biomolecular interactions [189]. These biosensors operate on the principle of modifying the transmission or reflection of light when target molecules bind to the sensor surface. Photonic crystals are engineered materials with periodic structures that create band gaps in the electromagnetic spectrum (Figure 9c). These band gaps prevent the propagation of certain wavelengths of light, thus resulting in specific optical properties. When biomolecules bind to the sensor surface, they cause changes in the refractive index or the dielectric environment. This perturbation affects the photonic band gap, thereby leading to light transmission or reflection alterations. These shifts are then used to quantify the presence or concentration of the target analyte.

Interferometric biosensors utilize the interference patterns generated when light waves interact. By measuring changes in the phase or intensity, these sensors detect biomolecular interactions. Fabry–Perot interferometers and Mach–Zehnder interferometers (see Figure 9d) are commonly used in this category. A Fabry–Perot interferometer exploits multiple-beam interference within a resonant optical cavity to precisely measure the wavelengths of light. It consists of two parallel mirrors with a small separation distance, thus creating a resonant cavity. When light is introduced into the cavity, it reflects repeatedly between these mirrors, thereby leading to constructive and destructive interference between the multiple reflected beams. Constructive interference enhances the intensity of light at specific wavelengths, while destructive interference reduces it at others, thus producing a pattern of interference fringes. By analyzing these fringes and their variations, Fabry–Perot interferometers can be used to determine the wavelengths of light and facilitate high-resolution spectral analysis. Mach–Zehnder interferometers are typically used in integrated optical biosensors. They consist of two parallel waveguides; one is exposed to the sample, and the other serves as a reference. Biomolecular interactions on the sample waveguide cause changes in the optical path length, thus leading to interference patterns that can be used to quantify the interactions. Interferometric biosensors have applications in medical diagnostics and environmental monitoring.

Optical fiber biosensors employ optical fibers as a core component for detecting and quantifying biological or chemical substances. These sensors are characterized by their capacity to harness light transmission through optical fibers for sensitive and real-time detection. The basic operation typically involves recognition elements, such as antibodies, enzymes, or other bioactive molecules, which are immobilized on the fiber’s surface. When the target analyte binds to this recognition element, it changes the fiber’s optical properties, such as light intensity, wavelength, or polarization. These changes are then quantified and correlated to the concentration of the target analyte. These sensors are compact, versatile, immune to electromagnetic interference, and suitable for remote sensing.

Organic bioelectronic optical sensors offer several advantages, including label-free detection, high sensitivity, rapid response times, and the potential for miniaturization and integration with other electronic components. As organic bioelectronics research advances, the further research and development of novel organic materials and innovative sensing platforms are expected to drive progress in optical sensing and its applications in various scientific and technological domains.

### 5.3. Piezoelectric Sensing

Piezoelectric biosensing is a powerful and versatile real-time mechanism that is used to detect and quantify biomolecular interactions. This sensing mechanism leverages the piezoelectric effect of certain materials, such as quartz or piezoelectric polymers, to transduce biomolecular binding events into measurable electrical signals. These mass-based biosensors are widely used in biomedical research, diagnostics, and pharmaceutical development due to their label-free, sensitive, and rapid detection capabilities.

The fundamental principle behind piezoelectric biosensing lies in the piezoelectric materials’ ability to convert mechanical stress into electrical signals. The biosensing platform typically consists of a piezoelectric transducer, such as a quartz crystal microbalance (QCM) or a piezoelectric polymer-coated cantilever, which is functionalized with specific biorecognition elements [192,193]. These biorecognition elements, such as antibodies, DNA, or enzymes, are carefully immobilized on the surface of the piezoelectric material. When the biosensing platform comes into contact with a biological sample, such as a solution containing biomolecules of interest (e.g., proteins, DNA, or antigens), the biorecognition elements interact selectively with the target biomolecules. This interaction leads to the formation of biomolecular complexes, thereby causing an increase in the mass or stiffness of the layer attached to the piezoelectric material.

**Figure 10:**
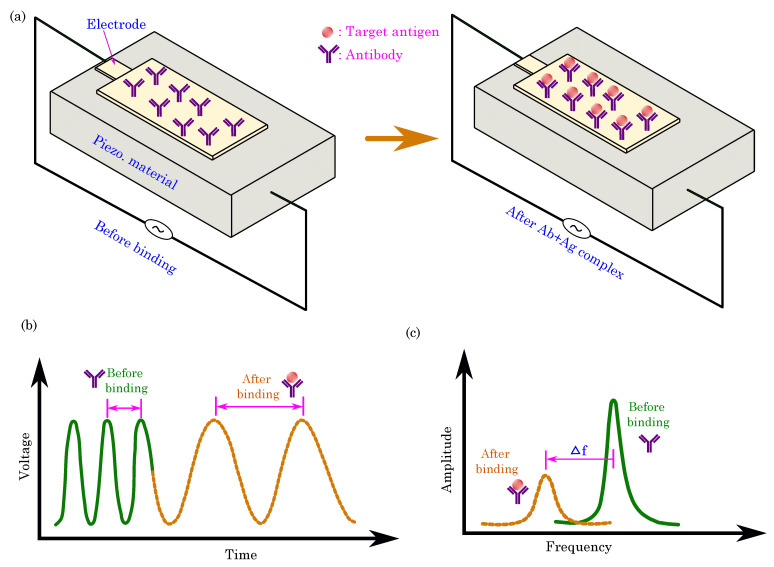
(**a**) Basic concept of target antigen detection mechanism using piezoelectric biosensing, (**b**) schematics of voltage vs. time, and (**c**) amplitude vs. frequency plots during detection.

As the biomolecular complexes form, the mechanical stress on the piezoelectric material changes, thereby inducing a shift in the resonant frequency of the piezoelectric transducer [194]. This frequency shift is directly proportional to the mass or stiffness change on the transducer’s surface and is known as the frequency shift or resonance frequency shift. The piezoelectric material converts this mechanical deformation into an electrical signal, thus generating a characteristic impedance change or charge distribution on the electrode surfaces. Figure 10 shows the basic concept of piezoelectric-sensor-based virus detection.

The interaction between the biorecognition elements and target biomolecules can be quantified and analyzed by monitoring the real-time frequency shift or electrical signals. This label-free detection approach directly measures biomolecular interactions without fluorescent or radioactive labels, which can alter biomolecules’ behavior and affect the measurements’ accuracy. Piezoelectric biosensors offer several advantages in bioanalytical applications, including higher sensitivity, real-time monitoring, label-free sensing, and multiplexing, thus enabling simultaneous detection of multiple target biomolecules in a single experiment, and they require low sample volumes, which make them suitable for analyzing limited or precious samples.

## 6. Biosensing Applications

### 6.1. Medical Diagnostics

Organic bioelectronics have emerged as a promising technology in medical diagnostics, thus offering unique advantages for noninvasive and point-of-care testing. By leveraging organic materials’ electrical and biological properties, organic bioelectronics facilitates the development of sensitive, portable, and cost-effective diagnostic devices [195,196,197].

Organic bioelectronic biosensors have opened up new possibilities in disease biomarker detection, thereby enabling the identification of specific biomolecules in biological fluids like blood, saliva, and urine [198,199]. These biosensors can be customized to detect disease-related biomarkers associated with conditions such as cancer, cardiovascular disorders, and infectious diseases, thereby facilitating early diagnosis and timely intervention. In the realm of diagnostics, organic bioelectronics play a central role in the miniaturization of diagnostic platforms, thus giving rise to lab-on-a-chip (LOC) devices [200,201]. LOC diagnostics offer rapid and multiplexed testing with minimal sample volume requirements, thus making them ideal for point-of-care settings and reducing the strain on centralized healthcare facilities. The use of organic bioelectronics extends to electrochemical and electronic immunoassays, thus providing highly sensitive and specific detection of antigens and antibodies. These assays allow for the precise quantification of disease-related molecules, thus supporting accurate diagnosis and monitoring of disease progression. Nucleic acid analysis is another application of organic bioelectronics, thereby enabling the detection of DNA and RNA sequences associated with genetic disorders and infectious agents [202,203]. This technology is essential for genetic screening, personalized medicine, and pathogen identification. In medical imaging, organic bioelectronics have shown promise in developing imaging probes and contrast agents, thereby enhancing the resolution and sensitivity of imaging techniques like magnetic resonance imaging (MRI) [204,205]. Additionally, organic bioelectronics have contributed to advancing microfluidic systems for cell analysis, thereby enabling cell sorting, counting, and characterizing cellular responses to external stimuli [206,207,208]. These systems have diverse applications in cancer diagnostics, drug screening, and stem cell research.

Furthermore, the potential for smart drug delivery systems arises from organic bioelectronics, thereby allowing for targeted drug delivery that responds to specific biological signals or conditions, as well as enhancing drug efficiency while minimizing side effects. Also, the portability and affordability of organic bioelectronic devices have made them a viable option for point-of-care diagnostics in resource-limited settings, offering timely and reliable medical testing in underserved regions. Altogether, organic bioelectronics are proving to be a transformative technology in medical and environmental applications, thereby contributing to improved healthcare, diagnostics, and research endeavors.

**Figure 11:**
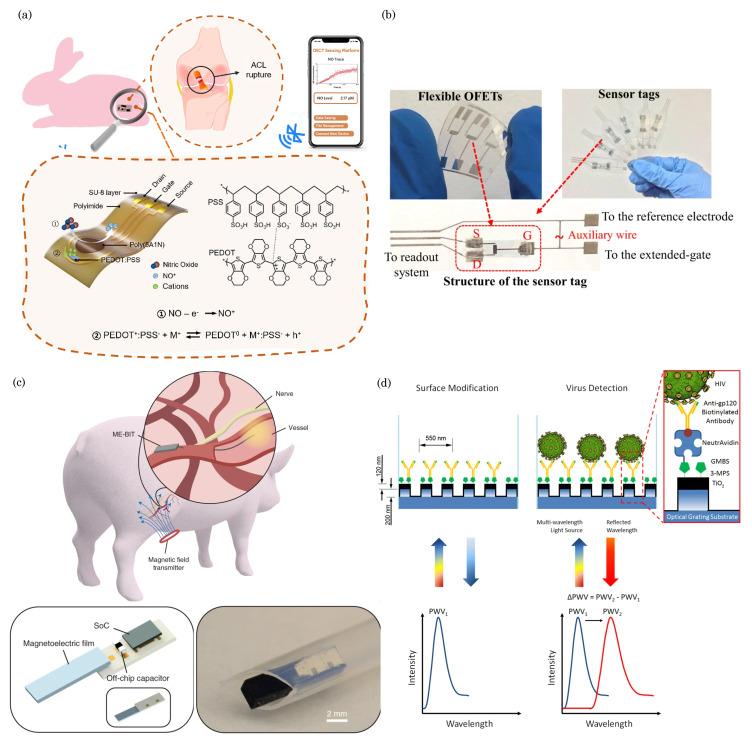
(**a**) Schematic illustration of a flexible OECT biosensor with wireless integration for real-time NO detection in an articular cavity. The NO sensor features a PEDOT:PSS channel, Au thin-film electrodes (source, drain, gate), poly-5A1N selective membrane on the gate, and SU-8 encapsulation exposing specific regions on a PI substrate. NO-induced electrochemical reactions on the gate electrode modulate PEDOT:PSS channel doping, thereby enabling NO sensing via current measurements. Implanted in a New Zealand white rabbit with ACL rupture, the sensor provided real-time NO monitoring by transmitting data to a mobile phone via a Bluetooth-enabled custom wireless module. Deng et al. [209], ©2022 the Author(s), licensed under Creative Commons Attribution-NonCommercial-NoDerivatives 4.0 International (CC BY-NC-ND 4.0). (**b**) Photo images of the fabricated low-voltage OFET miRNA sensor on PEN substrate, as well as the sensor tags consisting of an encapsulated OFET and contacts for extended-gate-sensing electrode and reference electrode. Reprinted from Tang et al. [210], licensed under a Creative Commons Attribution 4.0 International License. (**c**) Specific illustration of MagnetoElectric-powered Bio ImplanT (ME-BIT) device implanted proximally to a blood vessel deep within tissue and wirelessly powered through a magnetic coil in a pig. A rendering of the implant (**bottom left**) is shown with all the external components, including the system on a chip (SoC), external capacitor, and the ME transducer. Photograph of the fully packaged device inside a 3D-printed capsule resting in a clear sheath (**bottom right**). Reprinted with permission from Chen et al. [211], licensed under a Creative Commons Attribution 4.0, (**d**) Nanostructured optical photonic crystal biosensor for HIV viral load measurement. Reprinted with permission from Shafiee et al. [212], licensed under a Creative Commons Attribution-NonCommercial-NoDerivs 3.0 Unported License.

Figure 11 displays the diverse applications of organic bioelectronics in the field of medical diagnostics. Deng et al. [209] introduced a wireless, flexible, and highly sensitive biosensor employing organic electrochemical transistors (OECTs) for continuous and wireless nitric oxide (NO) detection within biological systems. Their OECT device, depicted in Figure 11, incorporated a PEDOT:PSS channel, gold (Au) thin-film electrodes (source, drain, and gate), a poly-5A1N-coated gate, and electrical contacts on a polyimide (PI) substrate. This sensor was successfully implanted in a rabbit for real-time NO monitoring, with the data transmitted wirelessly to a mobile phone via a custom Bluetooth module. Tang et al. [210] developed a low-power organic field-effect transistor (OFET)-based biochemical sensor with high transconductance efficiency for label-free miR-21 detection, as seen in Figure 11b. Additionally, Chen et al. [211] presented a compact wireless magnetoelectric endovascular neural stimulator that is illustrated in Figure 11c, which was specifically designed for battery-free implants, thus enabling the stimulation of peripheral nerves that are typically challenging to access via traditional surgical means.

### 6.2. Wearable Health Monitors

Organic bioelectronics have gained considerable traction as a technology for wearable health monitoring systems, thereby offering exceptional versatility and performance. Wearable devices can seamlessly integrate into daily life by leveraging organic materials’ unique properties, including flexibility, biocompatibility, and tunable electronics [213,214]. Applying organic bioelectronic sensors allows for the continuous and noninvasive monitoring of vital signs, such as heart rate [215], blood pressure [216], respiration rate [217,218], body temperature [219], pulse [220], glucose levels in individuals with diabetes [221], pH levels [222], and the human stress hormone cortisol [223]. Also, organic wearable bioelectronics have been widely used for chronic wound biosensing and on-demand therapy administration [224,225].

Furthermore, organic bioelectronics enable the recording of electrocardiogram (ECG) signals for the early detection of cardiac abnormalities while monitoring skin conditions, muscle activity during physical activities, sleep patterns, stress levels, and emotions, thereby contributing to a comprehensive health assessment [226,227,228]. These wearable systems can also track environmental factors like air quality and temperature, as well as provide secure biometric authentication for enhanced data security. By combining diverse functionalities, organic bioelectronics empower individuals to control their health proactively, thus enabling real-time remote monitoring, personalized drug delivery, and improved overall health management and outcomes [229,230]. As research continues, further advancements in organic bioelectronics promise to revolutionize wearable health monitoring technology and its potential impact on healthcare.

**Figure 12:**
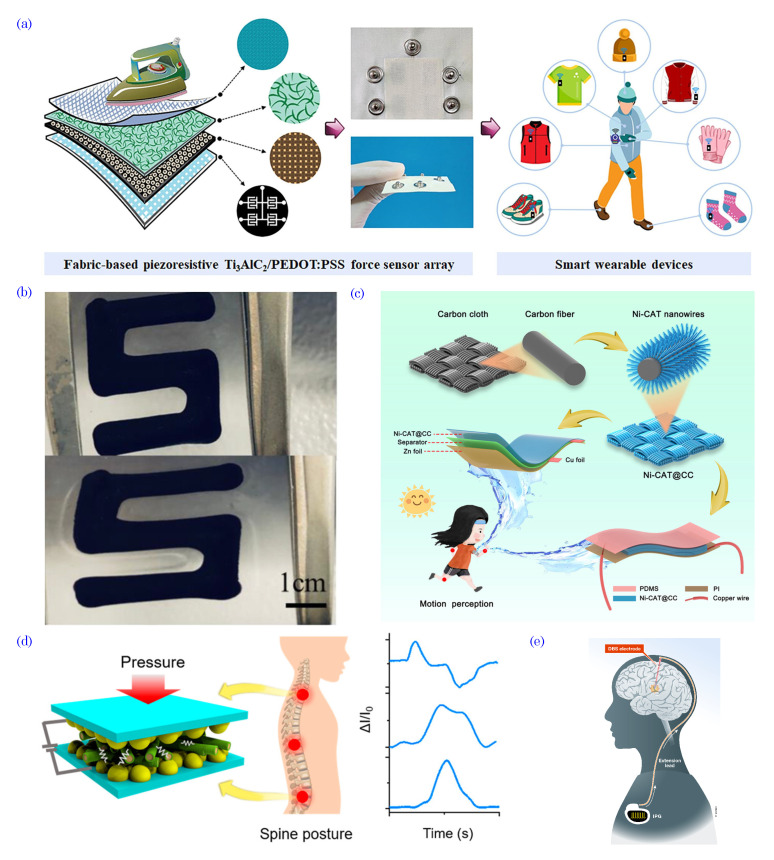
Examples of organic-bioelectronic-based sensors for wearable health monitoring applications. (**a**) Fabric-based piezoresistive force sensor array based on Ti3AlC2/PEDOT:PSS nanocomposite for wearable e-textile applications. Reprinted with permission from Seesaard and Wongchoosuk [231]. ©2023 Elsevier B.V. (**b**) Photographs of a stretchable photodiode made of a composite light absorber (P3HT:PCBM:SIS = 1:1:5) on a PDMS substrate before and after being stretched to about 25% strain. Adapted with permission from Mao et al. [232], ©2023 American Chemical Society. (**c**) Illustration depicting nickel-based metal–organic framework (MOF) nanowires employed as dual-purpose electrodes in wearable pressure sensor technology. Reprinted with permission from Fan et al. [233], ©2023 The Author(s). (**d**) Hierarchically microstructure-bioinspired flexible piezoresistive sensor or human–machine interaction and human health monitoring. The sensor incorporates a hierarchical polyaniline/polyvinylidene fluoride nanofiber (HPPNF) film positioned between two interlocking electrodes featuring a microdome structure. Reprinted with permission from Yang et al. [234], ©2021 American Chemical Society. (**e**) Schematic diagram for clinical application of deep brain stimulation (DBS) system: The brain electrode delivers therapeutic electrical currents, while the extension lead links it to the neurostimulator (internal pulse generator, IPG), which serves as the implanted power source. Reprinted with permission from Jacobs et al. [235], ©2019 The Author(s), published under the terms of the CC BY 4.0 license.

Figure 12 exemplifies applications of organic bioelectronics in wearable health monitoring and neuromodulation. Seesaard and Wongchoosuk [231] introduced a fabric-based piezoresistive force sensor array composed of a Ti${}_{3}$AlC${}_{2}$/PEDOT:PSS nanocomposite with ultrahigh sensitivity (up to 1.51 N${}^{-1}$) that is suitable for wearable e-textile applications. In another study, Mao et al. [232] developed a soft, stretchable photodiode with a composite light absorber and an organic bulk heterojunction within an elastic polymer matrix for reliable cardiovascular variable measurements. The developed photodiode effectively monitors variables such as heart rate variability and oxygen saturation over extended periods. Fan et al. [233] fabricated flexible wearable pressure sensors using free-standing conductive nickel metal–organic framework nanowire arrays on carbon cloth. The developed sensor could monitor human activities, including elbow, knee, and wrist bending, as illustrated in Figure 12c. Yang et al. [234] designed a flexible piezoresistive sensor with a hierarchical polyaniline/polyvinylidene fluoride nanofiber film for monitoring physiological signals and movement (see Figure 12d). Additionally, organic bioelectronics have found application in deep brain stimulation (DBS) for neuromodulation in movement disorders, such as Parkinson’s disease, where they connect brain electrodes to neurostimulators for therapeutic purposes, as depicted in Figure 12e.

### 6.3. Environmental Monitoring

Organic bioelectronics have demonstrated significant promise across diverse environmental monitoring applications due to their unique attributes, cost-effectiveness, and compatibility with biological systems. These applications achieve more efficient and sustainable monitoring solutions by leveraging organic electronic devices. Key areas of organic bioelectronics applications in environmental monitoring include water quality management and monitoring, thereby enabling the real-time detection of various pollutants in water bodies; air quality monitoring to track air pollution levels continuously; and soil health assessment, which all aid in precision agriculture.

In water quality management, organic bioelectronics are crucial in detecting and quantifying water pollutants such as heavy metals, organic compounds, and microorganisms [236,237]. Organic bioelectronic sensors offer high sensitivity and selectivity, thereby enabling real-time water quality monitoring in lakes, rivers, and wastewater treatment facilities [238,239]. These sensors can help identify contamination sources, assess the effectiveness of water treatment processes, and ensure compliance with regulatory standards, thereby preserving water resources and safeguarding aquatic ecosystems.

Similarly, organic bioelectronic sensors can assess essential parameters such as nutrient levels, pH, moisture content, and contaminants in soil quality monitoring [240,241]. Continuous soil monitoring using these sensors aids in precision agriculture, thus optimizing fertilizer usage, improving crop yield, and preventing soil degradation. By providing accurate and timely data on soil health, organic bioelectronics support sustainable land management practices and agriculture waste management, as well as promote soil conservation [242]. Additionally, organic bioelectronics are utilized for gas sensing, including greenhouse gases and harmful substances, which are critical for climate change studies and emissions control. For example, Alizadeh and colleagues introduced a molecularly imprinted polymer (MIP)-based electrochemical sensor designed to detect 2,4,6-trinitrotoluene (TNT) in environmental samples such as natural waters and soil [243]. The sensor operates using electrochemical principles, where the interaction between the imprinted polymer and TNT molecules leads to changes in the sensor’s electrical properties. Electrochemical techniques can measure and quantify this interaction, thereby offering a sensitive and reliable means of detecting TNT.

Moreover, integrating organic bioelectronics into wearable devices enables individuals to monitor personal exposure to environmental pollutants and allergens, thereby facilitating informed decisions to minimize exposure risks. These sensors’ lightweight and portable nature also makes them ideal for monitoring environmental parameters in remote and challenging-to-access areas, thus making them valuable for ecological studies and conservation efforts. The ability to network organic bioelectronic devices creates real-time large-scale environmental monitoring networks, thus contributing to predictive modeling, early warning systems, and informed environmental management decisions. Moreover, organic bioelectronic biosensors offer the rapid and precise detection and quantification of water and soil contaminants, including pesticides and heavy metals, thus aiding analytical assessments of environmental samples. Applying organic bioelectronics in environmental monitoring demonstrates its potential to enhance environmental sustainability, advance ecological understanding, and drive effective decision–making in various domains.

**Figure 13:**
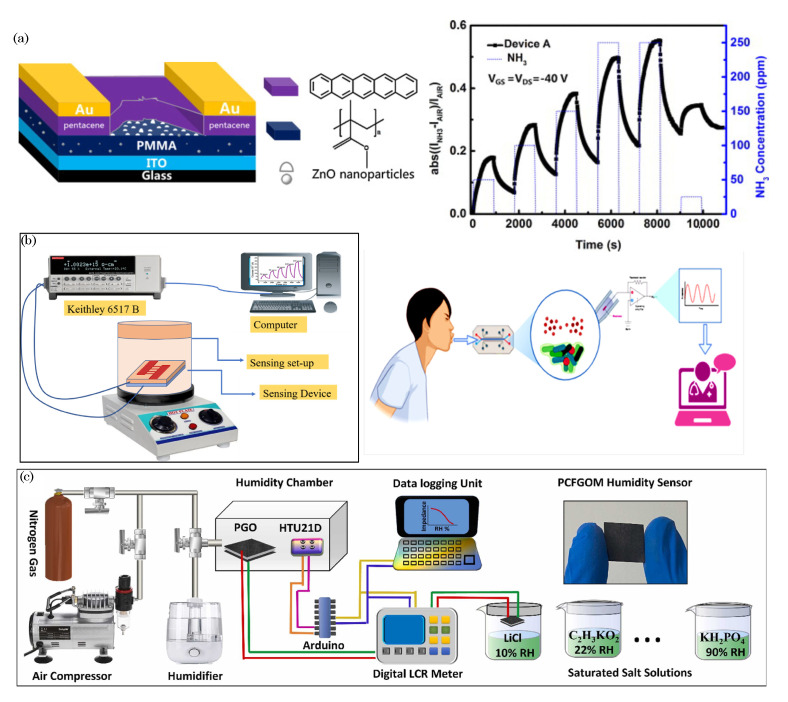
(**a**) Schematic structure of OFET biosensor for ammonia gas sensing (**left**). In this sensor, poly(methyl methacrylate) (PMMA) blended with zinc oxide (ZnO) nanoparticles is used as a gate dielectric layer. Response curves (**right**) of device’s (ZnO/PMMA hybrid dielectric) exposure to NH${}_{3}$ in higher concentrations (25–250 ppm). Reprinted with permission from Han et al. [244], ©2014 Elsevier B.V. (**b**) Schematic representation of the CuMoO${}_{4}$ nanorod−based acetone sensing measurement setup (**left**) and noninvasive breathomic diagnosis of human diabetes and environmental monitoring strategy (**right**). Reprinted with permission from Mathur et al. [246], ©2023 Elsevier Inc. (**c**) Biocompatible paper cellulose fiber graphene-oxide-matrix-based humidity sensors for human health and environment monitoring. Reprinted with permission from Khan et al. [245], ©2023 Elsevier B.V.

Figure 13 visually illustrates various organic bioelectronics sensors tailored for environmental monitoring applications. For example, Han et al. [244] introduced a highly efficient ammonia gas sensor by combining an organic field-effect transistor (OFET) with a ZnO/PMMA hybrid dielectric through a simple blending process. This sensor exhibited remarkable sensitivity across a wide range of NH${}_{3}$ concentrations, from 25 ppm to 250 ppm, as observed in Figure 13a, through the time-dependent changes in the drain–source current following multiple NH${}_{3}$ exposure and evacuation cycles. In a separate study, Mathur et al. fabricated CuMoO_4_ nanorods to create an acetone chemiresistor, thereby enabling noninvasive breath-based diabetes diagnosis and environmental monitoring (depicted in Figure 13b). Khan et al. [245] utilized a cellulose fiber and graphene oxide matrix to develop humidity sensors suitable for both environmental humidity monitoring and human respiration detection, as demonstrated in Figure 13c.

### 6.4. Food Safety and Quality Control

Organic bioelectronics has emerged as a promising technology for food safety and quality control applications [247,248,249,250]. Their unique properties, including biocompatibility and sensitivity to biological molecules, make them well suited for detecting contaminants, spoilage, and quality indicators in food products. Key applications include detecting food contaminants like pesticides and pathogens, monitoring food spoilage, assessing food quality indicators, and detecting allergens. Organic bioelectronics allows for real-time monitoring of food production processes and on-site testing, thus contributing to consistent quality and safety. Additionally, they can be integrated into smart packaging to monitor food quality during storage and transportation. This technology aids in verifying food authenticity, detecting adulteration, and ensuring agricultural production safety by monitoring pesticide residues on crops. Embracing organic bioelectronics in food safety and quality control enhances consumer protection, reduces food waste, and strengthens food safety regulations.

**Figure 14:**
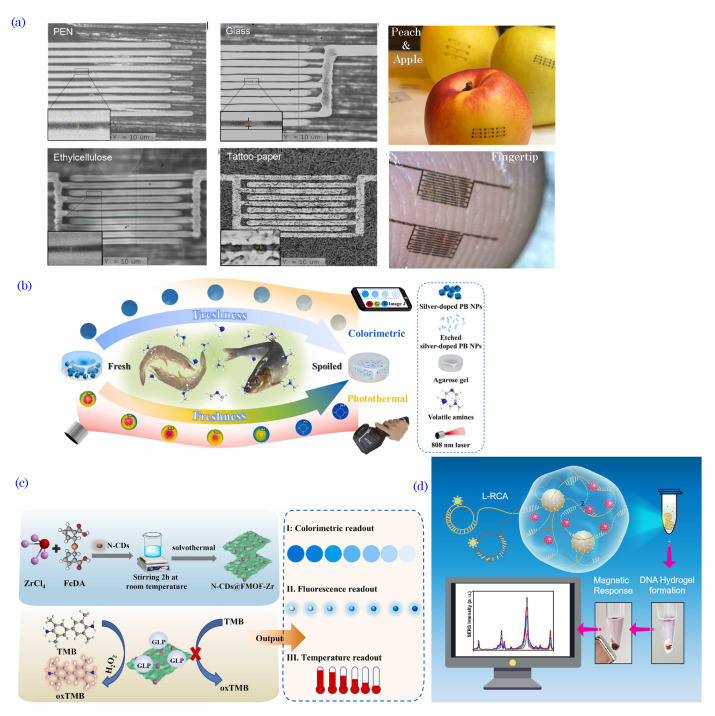
(**a**) Characterization of inkjet-printed gold electrodes on various conventional and edible substrates: Depiction of gold interdigitated electrodes inkjet-printed on diverse substrates: poly(ethylene 2,6-naphthalate) (PEN), glass, edible ethyl cellulose biopolymer (food additive E462), and edible tattoo paper. Visual representations of gold electrodes transferred onto distinct surfaces: (**right top**) peach, apple, and (**right bottom**) fingertip. Reprinted with permission from Sharova et al. [251], ©2023 The Royal Society of Chemistry. (**b**) Schematic representation of colorimetric and photothermal assessment of shrimp and fish freshness utilizing a portable silver-doped Prussian blue nanoparticles (SPB NPs) hydrogel, facilitated by a smartphone and handheld thermal imager. Reprinted with permission from Ding et al. [252], ©2022 Elsevier B.V. (**c**) Carbon dots anchoring ferrocene metal-organic framework nanosheet for multi-mode glyphosate (e.g., herbicide) sensing. Reprinted with permission from Luo et al. [253], ©2022 Elsevier B.V. (**d**) Schematic illustration of SERS aptasensor based on DNA hydrogel fishing network for ultrasensitive detection of antibacterial kanamycin (KANA). Reprinted with permission from Chen et al. [254], ©2022 Elsevier B.V.

Figure 14 illustrates the diverse applications of biosensors in food safety and quality control. Sharova et al. [251] introduced a low-voltage edible electronic circuit, thus serving as an invaluable testbed for exploring nontoxic printable semiconductors within the domains of edible and bioelectronic technologies. Their work, presented in Figure 14a, showcased successful inkjet printing of water-based gold ink on both traditional and edible substrates, thereby achieving exceptional precision with critical lateral features as small as 10 μm. Furthermore, they demonstrated the fabrication of chitosan-gated complementary n- and p-type transistors and logic circuits, including inverting logic gates, all operating at low voltages (<1 V) on flexible edible ethyl cellulose substrates. These devices exhibited promising electronic performance characteristics, such as high mobility–capacitance production, impressive on–off current ratios, operational stability in ambient air, and a shelf life of up to one month. These devices’ compact, flexible nature allows seamless integration into edible carriers, such as pharmaceutical capsules. In a separate study, Ding et al. [252] introduced a hydrogel containing silver-doped Prussian blue nanoparticles (SPB NPs) for the detection of trimethylamine (TMA) and the real-time monitoring of shrimp and fish freshness, as depicted in Figure 14b. Additionally, Luo et al. [253] explored using carbon dots anchored to ferrocene metal–organic framework nanosheets for the multimode sensing of glyphosate, which is a herbicide. In another application, Chen et al. [254] employed a DNA hydrogel fishing network for the ultrasensitive detection of the antibacterial agent kanamycin. These diverse applications underscore the remarkable versatility and potential of biosensors in enhancing food safety and quality control.

## 7. Challenges and Future Perspectives

### 7.1. Stability and Longevity

Stability and longevity are paramount considerations regarding applying organic electronics in biosensing, given their unique properties, such as flexibility and biocompatibility [255]. However, certain challenges contribute to potential degradation and performance fluctuations over time. First, organic materials are susceptible to environmental factors like moisture, oxygen, and temperature variations, thus leading to material degradation and subsequent changes in their electrical properties, thereby diminishing sensor performance [256]. Second, ensuring long-term biocompatibility when these devices interact with biological samples is critical to avoiding adverse reactions and preserving reliable sensing capabilities [257]. Third, the stability of the interface between the organic material and biomolecules significantly impacts biosensor performance, with changes from material degradation or biofouling affecting sensitivity and selectivity [258]. In wearable or implantable devices, the organic materials must endure mechanical stress without functional compromise, and mechanical strain may cause cracks or delamination, thereby jeopardizing stability and longevity [259]. Additionally, variations in performance over time due to charge trapping, ion migration, and relaxation processes can lead to sensor response drift, thus hampering accuracy. Sensitivity to chemicals and solvents can also affect stability and performance, which is a critical concern in real-world applications where chemical exposure is expected.

Moreover, organic materials may experience photochemical degradation when exposed to light, especially UV radiation, thereby impacting their electrical properties and sensor performance [260]. Finally, achieving manufacturing consistency and uniformity in organic electronic devices presents challenges, as variations in fabrication processes may lead to device-to-device performance differences, thereby influencing reproducibility and reliability. Addressing these stability and longevity concerns is essential for enhancing organic electronics’ long-term viability and effectiveness in biosensing applications.

Several strategies can be employed to address the stability and longevity issues associated with organic electronics in biosensing. These include careful material selection, implementing encapsulation techniques and barrier layers to protect the devices from environmental factors, optimizing device design for mechanical robustness, and performing rigorous testing and validation under relevant environmental conditions. Additionally, surface modifications and integrated control systems can enhance organic biosensors’ stability and operational performance. Overall, addressing these challenges will pave the way for the successful integration of organic electronics into cutting-edge biosensing technologies, thereby enabling advancements in medical diagnostics, environmental monitoring, and other critical applications.

### 7.2. Biocompatibility, Biofouling, and Cross-Sensitivity

Biocompatibility and biofouling pose significant challenges when utilizing organic electronics in biosensing applications. Despite the advantages of organic materials, such as flexibility and tunable properties, ensuring their compatibility with biological systems and mitigating the impact of biofouling is critical for reliable and long-term biosensor performance. In the realm of biocompatibility challenges, implantable biosensors necessitate favorable interactions between organic electronic materials and surrounding tissues to avoid inflammation or immune responses that may compromise the biosensor’s functionality and lifespan. Issues like cytotoxicity and impaired cell adhesion when in contact with biological fluids can disrupt stable biomolecule interactions, thereby leading to unreliable measurements. Additionally, an inflammatory response triggered by organic materials could result in encapsulation or scarring around the biosensor, thus hindering target analyte diffusion and affecting sensor sensitivity [261].

Moreover, leaching specific molecules from organic materials into the biological environment may compromise the biosensor’s accuracy and specificity. On the other hand, biofouling challenges encompass the non-specific binding of biomolecules, proteins, or cells to the biosensor surface, thereby generating unwanted signals and reducing sensitivity [262]. The accumulation of biofilm or organic material on the sensor surface can alter the electrical properties of the organic material, thus leading to a decline in sensor performance over time. Moreover, biofouling can hinder the diffusion of target analytes to the sensing elements, thus causing delayed or inaccurate readings and impacting the biosensor’s response time. Tackling these biocompatibility and biofouling challenges requires careful material selection, surface modifications, and the continuous research and development of innovative strategies to ensure the successful integration of organic electronics in biosensing applications.

Furthermore, cross-sensitivity within the domain of organic bioelectronics encompasses a significant challenge whereby sensors and devices, originally engineered to discern and respond to specific target analytes, also manifest responses to unintended analytes, thereby introducing ambiguity and inaccuracies into the device’s output. This pervasive issue permeates throughout the sensor and biosensor realm, including the specialized domain of organic bioelectronics. The implications of cross-sensitivity are noteworthy, thus encompassing potential distortions or falsifications of data, thereby diminishing the overall precision and reliability of the sensor. Several intricate factors contribute to cross-sensitivity in the context of organic bioelectronics. Firstly, material interactions stemming from the inherent properties of organic materials utilized in bioelectronic devices can predispose them to interactions with multiple analytes, which are exemplified by conducting polymers that may exhibit sensitivity to variances in pH, humidity, or temperature, thereby potentially fostering cross-sensitivity unless these issues are meticulously mitigated. Secondly, the propensity for analytes with resembling properties to induce overlapping sensor responses poses a significant challenge. For instance, two distinct gases may evoke analogous alterations in electrical conductivity, thus complicating their differentiation. Thirdly, the adsorption characteristics of the sensor’s surface may occasion unforeseen interactions with analytes, particularly in sensors reliant on specific binding events, such as antibody–antigen interactions. This can give rise to crossreactivity when analytes bearing similar structural or property traits adhere to the sensor’s surface. Lastly, environmental variables, including shifts in temperature, humidity, or interference from electromagnetic fields, may influence the sensor’s response, thereby potentially culminating in unwanted noise or disruptions that aggravate cross-sensitivity concerns. Cross-sensitivity challenges require meticulous consideration when designing, implementing, and employing organic bioelectronic devices, particularly in mission-critical applications such as medical diagnostics and environmental monitoring, where precision and fidelity are indispensable.

Researchers and engineers have explored various strategies to address the biocompatibility and biofouling challenges associated with organic electronics in biosensing [263,264]. Surface engineering techniques, such as functionalization with biocompatible coatings or polymers, enhance the biocompatibility of organic materials and reduce nonspecific binding [265,266]. Implementing biocompatible encapsulation materials or membranes isolates the organic electronics from direct contact with biological fluids, thereby minimizing adverse tissue interactions. Coating the sensor surface with antifouling agents prevents the adhesion of biomolecules and reduces the impact of biofouling on sensor performance. Rigorous in vitro and in vivo biocompatibility testing are crucial to identify potential cytotoxicity or inflammatory responses early in development. Employing regeneration methods, such as chemical or enzymatic cleaning, helps restore sensor functionality and combat the effects of biofouling. The continual research and development of new organic materials with improved biocompatibility and resistance to biofouling are essential to advance organic electronics for biosensing applications. Additionally, addressing cross-sensitivity in organic bioelectronics necessitates a multifaceted approach, spanning judicious material selection, intelligent surface functionalization strategies, advanced data processing techniques, and rigorous calibration measures to rectify inaccuracies arising from environmental factors. This multipronged strategy not only underscores the complexity of addressing cross-sensitivity in organic bioelectronics, but also highlights the necessity for a holistic and integrated approach, where material science, surface engineering, advanced data analysis, and robust calibration regimes converge to mitigate the challenges posed by cross-sensitivity, thereby ultimately contributing to the enhanced accuracy and reliability of organic bioelectronic devices.

By addressing these challenges, researchers can enhance the reliability and longevity of organic electronic biosensors, thereby paving the way for their successful integration in a wide range of biosensing applications, from medical diagnostics to environmental monitoring and beyond.

### 7.3. Manufacturing Scalability

Manufacturing scalability poses a critical challenge in utilizing organic electronics for biosensing applications despite the advantages of flexibility and cost-effectiveness offered by organic materials. The endeavor to achieve large-scale and reproducible manufacturing encounters several obstacles. These include maintaining material consistency to ensure uniform sensor characteristics and reliable performance, thereby tackling challenges in scaling up deposition techniques like inkjet printing and spin coating while preserving sensor integrity and addressing the complexities of device integration with multiple functional layers. Moreover, managing yield and reproducibility risks, achieving cost-effectiveness, and ensuring stability and reliability in large-scale production are paramount. Robust quality control measures are indispensable for the early identification and resolution of manufacturing issues, thereby encompassing material testing, sensor characterization, and performance validation. A reliable supply chain for high-quality organic materials is also crucial for sustained sensor performance and product reliability in the realm of organic electronics biosensing.

To address the manufacturing scalability challenges related to organic electronics in biosensing, researchers and industry stakeholders are exploring various approaches. Developing innovative and scalable manufacturing techniques, such as lithography, roll-to-roll printing, and spray coating, can improve production efficiency and material utilization [267,268]. Establishing standardized protocols and optimizing manufacturing processes can enhance yield, reproducibility, and material consistency. Integrating real-time quality control measures during manufacturing can detect deviations and ensure uniform sensor performance. Conducting rigorous, long-term stability testing under various environmental conditions is crucial to assessing sensor performance and reliability over extended periods. The widespread adoption of organic electronics in biosensing applications can be realized by tackling these obstacles, thereby paving the way for developing cost-effective, high-performance biosensors capable of transforming healthcare, environmental monitoring, and other critical domains.

### 7.4. Integration and Miniaturization

Incorporating organic bioelectronics into biosensing devices poses significant challenges in integration and miniaturization. Although organic materials offer unique advantages like flexibility and biocompatibility, achieving seamless integration into compact and multifunctional biosensors requires overcoming various obstacles. Key issues encompass multifunctional integration to create advanced biosensors capable of detecting multiple analytes and coordinating interactions between organic electronic components. Optimizing the sensor–substrate interface when integrating onto diverse substrates is essential to avoid performance degradation [269]. Power supply and energy efficiency become crucial in miniaturized biosensors operating on limited power sources [270]. Maintaining a high sensing performance and signal-to-noise ratio in shrinking biosensors is challenging due to signal interference and noise [271]. Precision in fabrication processes and high yield rates are crucial to achieving accurate dimensions and meeting demand while reducing production costs. Efficient data communication and onboard data processing are vital for real-time data transmission in miniaturized biosensors. Ensuring stability, longevity, enhanced biocompatibility, and addressing biofouling challenges are critical to maintaining reliable sensor performance over time in downsized organic bioelectronic components.

Researchers and engineers have employed various strategies to address integration and miniaturization challenges related to organic bioelectronics in biosensing. State-of-the-art microfabrication techniques enable precise control over sensor dimensions and facilitate multicomponent integration. Selecting suitable materials and optimizing sensor–substrate interfaces ensure compatibility and mechanical stability in miniaturized biosensors [258]. Designing low-power circuits and exploring energy-efficient strategies (e.g., self-powered sensors) extend miniaturized biosensors’ battery life and autonomy [272,273]. Implementing noise reduction techniques and signal amplification methods enhance the signal-to-noise ratio in miniaturized biosensors [274,275,276]. Utilizing automated manufacturing processes ensures reproducibility and precision, while robust quality control measures identify defects early in production. System-on-chip integration enables onboard data processing, thereby reducing the need for external data handling devices [277,278,279]. Applying biocompatible coatings to miniaturized biosensors improves biocompatibility and reduces biofouling [280,281,282]. Effectively addressing these challenges empowers organic bioelectronics to pave the way for highly compact and versatile biosensors with applications ranging from wearable health monitoring to point-of-care diagnostics, thereby advancing healthcare and biosensing capabilities.

### 7.5. Data Security and Privacy

Data security and privacy are crucial concerns in the context of using organic electronics in biosensing applications [283,284,285]. With sensitive biological and health-related data being collected by these devices, maintaining the confidentiality and integrity of this information becomes paramount. Key issues include securing data transmission through robust encryption protocols, authenticating the biosensing device and its generated data to prevent tampering and unauthorized access, and ensuring secure data storage with strong encryption and access control measures. It is also essential to implement secure communication protocols between the biosensor and external devices or servers, anonymize and de-identify collected data to protect individual privacy, and guard against cyberattacks like malware and ransomware [286,287]. Compliance with data protection regulations like GDPR and HIPAA is necessary, as is user awareness and education about data security best practices. A well-defined data breach response plan and proper data erasure procedures at the end of a device’s life cycle are additional measures to mitigate risks and ensure the ethical use of biosensor data. As organic electronics advance in biosensing, a comprehensive approach to data protection is essential to foster trust and safeguard sensitive information.

### 7.6. Future Perspectives of Organic Bioelctronics

Recent times have witnessed a revolutionary transformation in biosensor technology achieved through synergistic integration with cutting-edge technologies such as smartphones, 3D printing, artificial intelligence, and the Internet of Things (IoTs) [288]. This convergence has led to unprecedented advancements and opportunities in biosensors. By leveraging the capabilities of these emerging technologies, biosensors have become more accessible, versatile, and efficient than ever before. Smartphones now serve as portable and user-friendly interfaces for real-time data collection and analysis, thus making biosensing widely accessible. The field of 3D printing has enabled the rapid prototyping and customization of biosensors, thereby allowing tailored designs to meet specific application requirements. Artificial intelligence has empowered biosensors with advanced data processing and pattern recognition capabilities, enhancing accuracy and enabling predictive analytics. The IoTs has facilitated seamless connectivity and remote monitoring of biosensors, thereby enabling real-time data transmission and applications in remote and distributed environments. This amalgamation has opened new horizons in healthcare, environmental monitoring, food safety, and beyond, thus reshaping the future of biosensor applications.

Moreover, the trajectory of organic bioelectronics in intelligent biosensing strategies holds immense promise due to rapid technological progress and interdisciplinary collaborations. This trajectory envisions multiple transformative directions that underline the potential evolution of intelligent biosensing using organic bioelectronics. These include the development of smart biosensing platforms that can autonomously make decisions and incorporate artificial intelligence algorithms for real-time analyte detection and quantification [289]. Additionally, there’s a growing focus on sensors that can self-calibrate using internal or external reference signals to enhance accuracy and reliability [290,291,292]. Integrating data from different sensors employing diverse sensing modalities promises a more comprehensive understanding of sample composition. The concept of dynamic sampling, where sensors adapt their sampling rates based on detected analyte shifts, could optimize energy usage while ensuring timely detection. Furthermore, realizing interconnected sensing networks, predictive analytics, human–machine interfaces, personalized medical interventions, energy-efficient designs, and remote monitoring through telehealth services showcase the broad scope of organic bioelectronics’ role in revolutionizing intelligent biosensing [293].

Furthermore, the advancement of sustainable organic bioelectronic sensors holds significant promise, which is propelled by progress in materials science, biotechnology, and a growing environmental consciousness. These sensors are increasingly capable of utilizing biodegradable and environmentally friendly materials, thereby minimizing their ecological footprint [294]. The integration of energy-harvesting technologies further lessens their dependence on traditional batteries by tapping into renewable sources like solar energy or vibrations [295,296]. The potential for mass production of flexible and printable organic electronics opens doors to versatile applications, including healthcare and environmental monitoring. Moreover, affordable, sustainable bioelectronic sensors are pivotal in addressing global health challenges by facilitating remote disease monitoring in resource-limited regions.

## 8. Conclusions

Organic electronics in biosensing represent a promising and dynamic frontier with far-reaching implications for medical and environmental applications. This exciting convergence of organic materials and bioelectronics has unlocked new opportunities for the precise, sensitive, and real-time detection of biomolecules and chemical species, thereby transforming the landscape of medical diagnostics and environmental monitoring.

The unique properties of organic materials, such as biocompatibility, flexibility, and tunability, have paved the way for developing innovative biosensing devices with diverse applications. From implantable biosensors for continuous health monitoring to wearable devices enabling personalized diagnostics, organic bioelectronics offer groundbreaking solutions that bridge the gap between traditional sensing technologies and cutting-edge medical practices. In medical diagnostics, organic bioelectronic sensors offer the potential to revolutionize disease detection and management. These sensors’ label-free and real-time monitoring capabilities enable the rapid and accurate analysis of biomarkers, thereby facilitating early disease diagnosis and tailored treatment plans. Moreover, integrating organic bioelectronics into wearable health monitoring systems empowers individuals to actively participate in their healthcare, thereby promoting proactive and personalized health management. Beyond medical applications, the versatility of organic bioelectronics finds significant relevance in environmental monitoring. From detecting pollutants and toxins to monitoring changes in environmental parameters, organic bioelectronic sensors contribute to sustainable environmental management and conservation efforts. These sensors offer the potential for the rapid and efficient detection of environmental threats, thereby enabling timely interventions and preserving ecological balance. However, as with any emerging technology, organic electronics in biosensing face challenges that warrant attention. Issues related to biocompatibility, stability, scalability, and manufacturing consistency must be addressed to ensure these biosensing platforms’ reliability and long-term performance.

In conclusion, integrating organic electronics in biosensing is promising for medical and environmental applications. With ongoing research and collaborative efforts between scientists, engineers, and industry stakeholders, organic bioelectronics are poised to drive transformative advancements in healthcare and environmental sustainability. By harnessing the potential of organic materials and innovative sensing mechanisms, this frontier of biosensing promises to improve human health, protect the environment, and shape a more sustainable and technologically advanced future.

## Figures and Tables

**Table 1:** Summary of fabrication techniques for organic electronics.

Fabrication Techniques	Material	References
Spin coating	2D crystalline film from 2,7-diocty[1]benzothieno[3,2-b]benzothiophene (C8-BTBT), PDMS, organic semiconductor films, PEDOT:PSS	[145,146,147,148]
Photolithography	PEDOT:PSS, OLED	[124,149,150,151]
E-beam lithography	PPy, poly(chloro-p-xylylene) (Parylene C), biomolecules	[152,153,154,155]
Dip-pen nanolithography	sulfonated polyaniline (SPAN), PPy, PEDOT, ferroelectric copolymer poly (vinylidene fluoride– trifluorethylene)	[156,157]
Inkjet printing	PEDOT:PSS, PPy	[128,129,130,158]
Micro contact printing	PPy, PEDOT, proteins, ultrathin gate dielectrics, alkyl and fluoroalkylphosphonic acid	[159,160,161,162,163]
Laser writing	PEDOT, PANI, laser-induced porous graphene	[164,165]
Direct ink writing	PEDOT:PSS, PEDOT:PSS-PEO, holey graphene oxide (hGO), eutectic gallium–indium (EGaIn)-based liquid metal embedded elastomers, AgNPs, MWCNT, rGO/CNT, silicone	[166,167,168,169,170,171,172]
Chemical vapor deposition	Poly(p-xylylene), PEDOT	[173,174]

## Data Availability

Not applicable.

## References

[1] Le T.H., Kim Y., Yoon H. (2017). Electrical and electrochemical properties of conducting polymers. Polymers.

[2] Brédas J.L., Calbert J.P., da Silva Filho D., Cornil J. (2002). Organic semiconductors: A theoretical characterization of the basic parameters governing charge transport. Proc. Natl. Acad. Sci. USA.

[3] Park S., Kang Y.J., Majd S. (2015). A review of patterned organic bioelectronic materials and their biomedical applications. Adv. Mater..

[4] Havinga E., Ten Hoeve W., Meijer E., Wynberg H. (1989). Water-soluble self-doped 3-substituted polypyrroles. Chem. Mater..

[5] Wang Z., Zheng N., Zhang W., Yan H., Xie Z., Ma Y., Huang F., Cao Y. (2017). Self-Doped, n-Type Perylene Diimide Derivatives as Electron Transporting Layers for High-Efficiency Polymer Solar Cells. Adv. Energy. Mater..

[6] Powell D., Zhang X., Nwachukwu C.I., Miller E.J., Hansen K.R., Flannery L., Ogle J., Berzansky A., Labram J.G., Roberts A.G. (2022). Establishing Self-Dopant Design Principles from Structure–Function Relationships in Self-n-Doped Perylene Diimide Organic Semiconductors. Adv. Mater..

[7] Ramanavičius A., Ramanavičienė A., Malinauskas A. (2006). Electrochemical sensors based on conducting polymer—Polypyrrole. Electrochim. Acta.

[8] Paulsen B.D., Tybrandt K., Stavrinidou E., Rivnay J. (2020). Organic mixed ionic–electronic conductors. Nat. Mater..

[9] Lu Z., Pavia A., Savva A., Kergoat L., Owens R.M. (2023). Organic microelectrode arrays for bioelectronic applications. Mater. Sci. Eng. R Rep..

[10] Wu R., Matta M., Paulsen B.D., Rivnay J. (2022). Operando characterization of organic mixed ionic/electronic conducting materials. Chem. Rev..

[11] Brütting W. (2005). Physics of Organic Semiconductors.

[12] Köhler A., Bässler H. (2015). Electronic Processes in Organic Semiconductors: An Introduction.

[13] Kunkel C., Margraf J.T., Chen K., Oberhofer H., Reuter K. (2021). Active discovery of organic semiconductors. Nat. Commun..

[14] Chen J., Zhang W., Wang L., Yu G. (2023). Recent research progress of organic small-molecule semiconductors with high electron mobilities. Adv. Mater..

[15] Mishra A., Bäuerle P. (2012). Small molecule organic semiconductors on the move: Promises for future solar energy technology. Angew. Chem. Int. Ed..

[16] Coropceanu V., Cornil J., da Silva Filho D.A., Olivier Y., Silbey R., Brédas J.L. (2007). Charge transport in organic semiconductors. Chem. Rev..

[17] Sun Y., Liu Y., Zhu D. (2005). Advances in organic field-effect transistors. J. Mater. Chem..

[18] Zhang G., Xie C., You P., Li S. (2022). Organic field-effect transistors. Introduction to Organic Electronic Devices.

[19] Dodabalapur A. (1997). Organic light emitting diodes. Solid State Commun..

[20] Gather M.C., Köhnen A., Meerholz K. (2011). White organic light-emitting diodes. Adv. Mater..

[21] Song J., Lee H., Jeong E.G., Choi K.C., Yoo S. (2020). Organic light-emitting diodes: Pushing toward the limits and beyond. Adv. Mater..

[22] Brabec C.J. (2004). Organic photovoltaics: Technology and market. Sol. Energy Mater. Sol. Cells.

[23] Kippelen B., Brédas J.L. (2009). Organic photovoltaics. Energy Environ. Sci..

[24] Inganäs O. (2018). Organic photovoltaics over three decades. Adv. Mater..

[25] Wu Y.L., Fukuda K., Yokota T., Someya T. (2019). A highly responsive organic image sensor based on a two-terminal organic photodetector with photomultiplication. Adv. Mater..

[26] Nasri A., Petrissans M., Fierro V., Celzard A. (2021). Gas sensing based on organic composite materials: Review of sensor types, progresses and challenges. Mater. Sci. Semicond..

[27] Hopkins J., Fidanovski K., Lauto A., Mawad D. (2019). All-organic semiconductors for electrochemical biosensors: An overview of recent progress in material design. Front. Bioeng. Biotechnol..

[28] Borges-González J., Kousseff C.J., Nielsen C.B. (2019). Organic semiconductors for biological sensing. J. Mater. Chem. C.

[29] Ta J., Sun W., Lu L. (2022). Organic small molecule semiconductor materials for OFET-based biosensors. Biosens. Bioelectron..

[30] Kim K., Yoo H., Lee E.K. (2022). New Opportunities for organic semiconducting polymers in biomedical applications. Polymers.

[31] Subbarao N.V., Gedda M., Iyer P.K., Goswami D.K. (2016). Organic field-effect transistors as high performance humidity sensors with rapid response, recovery time and remarkable ambient stability. Org. Electron..

[32] Za’aba N.K., Morrison J.J., Taylor D.M. (2017). Effect of relative humidity and temperature on the stability of DNTT transistors: A density of states investigation. Org. Electron..

[33] Kimpel J., Michinobu T. (2021). Conjugated polymers for functional applications: Lifetime and performance of polymeric organic semiconductors in organic field-effect transistors. Polym. Int..

[34] Dong P., Yang L., Du G., Wang W., Rolston N., Zhang J. (2023). Anion-Modulated Chemical Doping of Organic Hole Conductor Boosts Efficiency and Stability of Perovskite Solar Cells. Adv. Funct. Mater..

[35] Neupane G.P., Ma W., Yildirim T., Tang Y., Zhang L., Lu Y. (2019). 2D organic semiconductors, the future of green nanotechnology. Nano Mater. Sci..

[36] Ranallo S., Bracaglia S., Sorrentino D., Ricci F. (2023). Synthetic Antigen-Conjugated DNA Systems for Antibody Detection and Characterization. ACS Sens..

[37] Kumar S., Bhushan P., Krishna V., Bhattacharya S. (2018). Tapered lateral flow immunoassay based point-of-care diagnostic device for ultrasensitive colorimetric detection of dengue NS1. Biomicrofluidics.

[38] Chaki N.K., Vijayamohanan K. (2002). Self-assembled monolayers as a tunable platform for biosensor applications. Biosens. Bioelectron..

[39] Sonawane M.D., Nimse S.B. (2016). Surface modification chemistries of materials used in diagnostic platforms with biomolecules. J. Chem..

[40] Sandhyarani N. (2019). Surface modification methods for electrochemical biosensors. Electrochemical Biosensors.

[41] Li L., Wang S., Xiao Y., Wang Y. (2020). Recent advances in immobilization strategies for biomolecules in sensors using organic field-effect transistors. Trans. Tianjin Univ..

[42] Rocchitta G., Spanu A., Babudieri S., Latte G., Madeddu G., Galleri G., Nuvoli S., Bagella P., Demartis M.I., Fiore V. (2016). Enzyme biosensors for biomedical applications: Strategies for safeguarding analytical performances in biological fluids. Sensors.

[43] Gonzalez-Gonzalez R.B., Flores-Contreras E.A., Gonzalez-Gonzalez E., Torres Castillo N.E., Parra-Saldivar R., Iqbal H.M. (2022). Biosensor constructs for the monitoring of persistent emerging pollutants in environmental matrices. Ind. Eng. Chem. Res..

[44] Mulchandani A., Mulchandani A., Rogers K. (1998). Principles of enzyme biosensors. Enzyme and Microbial Biosensors. Methods in Biotechnology.

[45] Zeng X., Shen Z., Mernaugh R. (2012). Recombinant antibodies and their use in biosensors. Anal. Bioanal. Chem..

[46] Sharma S., Byrne H., O’Kennedy R.J. (2016). Antibodies and antibody-derived analytical biosensors. Essays Biochem..

[47] Hahn S., Mergenthaler S., Zimmermann B., Holzgreve W. (2005). Nucleic acid based biosensors: The desires of the user. Bioelectrochemistry.

[48] Palchetti I., Mascini M. (2008). Nucleic acid biosensors for environmental pollution monitoring. Analyst.

[49] Du Y., Dong S. (2017). Nucleic acid biosensors: Recent advances and perspectives. Anal. Chem..

[50] Fu Z., Lu Y.C., Lai J.J. (2019). Recent advances in biosensors for nucleic acid and exosome detection. Chonnam Med. J..

[51] Kumar S., Bhushan P., Bhattacharya S., Bhattacharya S., Agarwal A., Chanda N., Pandey A., Sen A. (2018). Fabrication of nanostructures with bottom-up approach and their utility in diagnostics, therapeutics, and others. Environmental, Chemical and Medical Sensors—Energy, Environment, and Sustainability.

[52] Ramya M., Kumar P.S., Rangasamy G., Rajesh G., Nirmala K., Saravanan A., Krishnapandi A. (2022). A recent advancement on the applications of nanomaterials in electrochemical sensors and biosensors. Chemosphere.

[53] Jain P.K., Huang X., El-Sayed I.H., El-Sayed M.A. (2007). Review of some interesting surface plasmon resonance-enhanced properties of noble metal nanoparticles and their applications to biosystems. Plasmonics.

[54] Liu M., Mou J., Xu X., Zhang F., Xia J., Wang Z. (2020). High-efficiency artificial enzyme cascade bio-platform based on MOF-derived bimetal nanocomposite for biosensing. Talanta.

[55] Stephanie R., Kim M.W., Kim S.H., Kim J.K., Park C.Y., Park T.J. (2021). Recent advances of bimetallic nanomaterials and its nanocomposites for biosensing applications. TrAC Trends Anal. Chem..

[56] Kumar S., Bhushan P., Bhattacharya S. (2017). Facile synthesis of Au@ Ag–hemin decorated reduced graphene oxide sheets: A novel peroxidase mimetic for ultrasensitive colorimetric detection of hydrogen peroxide and glucose. RSC Adv..

[57] Tasis D., Tagmatarchis N., Bianco A., Prato M. (2006). Chemistry of carbon nanotubes. Chem. Rev..

[58] Maruyama T. (2021). Carbon nanotubes. Handbook of Carbon-Based Nanomaterials.

[59] Pumera M. (2011). Graphene in biosensing. Mater. Today.

[60] Kuila T., Bose S., Khanra P., Mishra A.K., Kim N.H., Lee J.H. (2011). Recent advances in graphene-based biosensors. Biosens. Bioelectron..

[61] Morales-Narváez E., Baptista-Pires L., Zamora-Gálvez A., Merkoçi A. (2017). Graphene-based biosensors: Going simple. Adv. Mater..

[62] Kumar S., Bhushan P., Bhattacharya S. (2016). Development of a paper-based analytical device for colorimetric detection of uric acid using gold nanoparticles–graphene oxide (AuNPs–GO) conjugates. Anal. Methods.

[63] Ghosal K., Sarkar K. (2018). Biomedical applications of graphene nanomaterials and beyond. ACS Biomater. Sci. Eng..

[64] Byakodi M., Shrikrishna N.S., Sharma R., Bhansali S., Mishra Y., Kaushik A., Gandhi S. (2022). Emerging 0D, 1D, 2D, and 3D nanostructures for efficient point-of-care biosensing. Biosens. Bioelectron. X.

[65] Chen M., Wang Y., Su H., Mao L., Jiang X., Zhang T., Dai X. (2018). Three-dimensional electrochemical DNA biosensor based on 3D graphene-Ag nanoparticles for sensitive detection of CYFRA21-1 in non-small cell lung cancer. Sens. Actuators B Chem..

[66] Chen M., Wu D., Tu S., Yang C., Chen D., Xu Y. (2021). CRISPR/Cas9 cleavage triggered ESDR for circulating tumor DNA detection based on a 3D graphene/AuPtPd nanoflower biosensor. Biosens. Bioelectron..

[67] Zhou Y., Lv S., Wang X.Y., Kong L., Bi S. (2022). Biometric photoelectrochemical–visual multimodal biosensor based on 3D hollow HCdS@ Au nanospheres coupled with target-induced ion exchange reaction for antigen detection. Anal. Chem..

[68] Ealia S.A.M., Saravanakumar M.P. (2017). A review on the classification, characterisation, synthesis of nanoparticles and their application. IOP Conf. Ser. Mater. Sci. Eng..

[69] Joudeh N., Linke D. (2022). Nanoparticle classification, physicochemical properties, characterization, and applications: A comprehensive review for biologists. J. Nanobiotechnol..

[70] Elsaesser A., Howard C.V. (2012). Toxicology of nanoparticles. Adv. Drug Deliv. Rev..

[71] Sengul A.B., Asmatulu E. (2020). Toxicity of metal and metal oxide nanoparticles: A review. Environ. Chem. Lett..

[72] Yang W., Wang L., Mettenbrink E.M., DeAngelis P.L., Wilhelm S. (2021). Nanoparticle toxicology. Annu. Rev. Pharmacol. Toxicol..

[73] Berggren M., Richter-Dahlfors A. (2007). Organic bioelectronics. Adv. Mater..

[74] Mei J., Diao Y., Appleton A.L., Fang L., Bao Z. (2013). Integrated materials design of organic semiconductors for field-effect transistors. J. Am. Chem. Soc..

[75] Horowitz G. (1998). Organic field-effect transistors. Adv. Mater..

[76] Picca R.A., Manoli K., Macchia E., Sarcina L., Di Franco C., Cioffi N., Blasi D., Österbacka R., Torricelli F., Scamarcio G. (2020). Ultimately sensitive organic bioelectronic transistor sensors by materials and device structure design. Adv. Funct. Mater..

[77] Zhang X., Pu Z., Su X., Li C., Zheng H., Li D. (2023). Flexible organic field-effect transistors-based biosensors: Progress and perspectives. Anal. Bioanal. Chem..

[78] Chan P.K.L. (2019). The Motivation for and Challenges to Scaling Down Organic Field-Effect Transistors. Adv. Electron. Mater..

[79] Wang C., Zhang X., Dong H., Chen X., Hu W. (2020). Challenges and emerging opportunities in high-mobility and low-energy-consumption organic field-effect transistors. Adv. Energy Mater..

[80] Ajayan J., Mohankumar P., Mathew R., Thoutam L.R., Kaushik B.K., Nirmal D. (2023). Organic Electrochemical Transistors (OECTs): Advancements and Exciting Prospects for Future Biosensing Applications. IEEE Trans. Electron Devices..

[81] Friedlein J.T., McLeod R.R., Rivnay J. (2018). Device physics of organic electrochemical transistors. Org. Electron..

[82] Ait Yazza A., Blondeau P., Andrade F.J. (2021). Simple approach for building high transconductance paper-based organic electrochemical transistor (OECT) for chemical sensing. ACS Appl. Electron. Mater..

[83] Bernards D.A., Malliaras G.G. (2007). Steady-state and transient behavior of organic electrochemical transistors. Adv. Funct. Mater..

[84] Rivnay J., Inal S., Salleo A., Owens R.M., Berggren M., Malliaras G.G. (2018). Organic electrochemical transistors. Nat. Rev. Mater..

[85] Chen S., Surendran A., Wu X., Lee S.Y., Stephen M., Leong W.L. (2020). Recent technological advances in fabrication and application of organic electrochemical transistors. Adv. Mater. Technol..

[86] Simon D.T., Gabrielsson E.O., Tybrandt K., Berggren M. (2016). Organic bioelectronics: Bridging the signaling gap between biology and technology. Chem. Rev..

[87] Mei T., Zhang H., Xiao K. (2022). Bioinspired artificial ion pumps. ACS Nano.

[88] Ohayon D., Inal S. (2020). Organic bioelectronics: From functional materials to next-generation devices and power sources. Adv. Mater..

[89] Isaksson J., Kjäll P., Nilsson D., Robinson N., Berggren M., Richter-Dahlfors A. (2007). Electronic control of Ca^2+^ signalling in neuronal cells using an organic electronic ion pump. Nat. Mater..

[90] Tybrandt K., Larsson K.C., Kurup S., Simon D.T., Kjäll P., Isaksson J., Sandberg M., Jager E.W., Richter-Dahlfors A., Berggren M. (2009). Translating electronic currents to precise acetylcholine–induced neuronal signaling using an organic electrophoretic delivery device. Adv. Mater..

[91] Proctor C.M., Slézia A., Kaszas A., Ghestem A., Del Agua I., Pappa A.M., Bernard C., Williamson A., Malliaras G.G. (2018). Electrophoretic drug delivery for seizure control. Sci. Adv..

[92] Simon D.T., Kurup S., Larsson K.C., Hori R., Tybrandt K., Goiny M., Jager E.W., Berggren M., Canlon B., Richter-Dahlfors A. (2009). Organic electronics for precise delivery of neurotransmitters to modulate mammalian sensory function. Nature Mater..

[93] Jonsson A., Song Z., Nilsson D., Meyerson B.A., Simon D.T., Linderoth B., Berggren M. (2015). Therapy using implanted organic bioelectronics. Sci. Adv..

[94] Poxson D.J., Karady M., Gabrielsson R., Alkattan A.Y., Gustavsson A., Doyle S.M., Robert S., Ljung K., Grebe M., Simon D.T. (2017). Regulating plant physiology with organic electronics. Proc. Natl. Acad. Sci. USA.

[95] Jakešová M., Sjöström T.A., Đerek V., Poxson D., Berggren M., Głowacki E.D., Simon D.T. (2019). Wireless organic electronic ion pumps driven by photovoltaics. NPJ Flex. Electron..

[96] Strakosas X., Seitanidou M., Tybrandt K., Berggren M., Simon D.T. (2021). An electronic proton-trapping ion pump for selective drug delivery. Sci. Adv..

[97] Cherian D., Armgarth A., Beni V., Linderhed U., Tybrandt K., Nilsson D., Simon D.T., Berggren M. (2019). Large-area printed organic electronic ion pumps. Flex. Print. Electron..

[98] Liu J., Wang Y., Wen H., Bao Q., Shen L., Ding L. (2020). Organic photodetectors: Materials, structures, and challenges. Solar Rrl..

[99] Cea C., Zhao Z., Wisniewski D.J., Spyropoulos G.D., Polyravas A., Gelinas J.N., Khodagholy D. (2023). Integrated internal ion-gated organic electrochemical transistors for stand-alone conformable bioelectronics. Nat. Mater..

[100] Yang D., Ma D. (2019). Development of organic semiconductor photodetectors: From mechanism to applications. Adv. Opt. Mater..

[101] Schmidt B., Ross R. (1983). Position-sensitive photodetectors made with standard silicon-planar technology. Sens. Actuators.

[102] Tull C., Iwanczyk J., Patt B., Vilkelis G., Eremin V., Verbitskaya E., Strokan N., Il’yashenko I., Ivanov A., Sidorov A. New high sensitivity silicon photodetectors for medical imaging applications. Proceedings of the 2002 IEEE Nuclear Science Symposium Conference Record.

[103] Caria M., Barberini L., Cadeddu S., Giannattasio A., Rusani A., Sesselego A., Lai A., D’Auria S., Dubecky F. (2002). Gallium arsenide photodetectors for imaging in the far ultraviolet region. Appl. Phys. Lett..

[104] Li J., Lu Q., Dai H., Chen Z., Fu Y., Chen X. (2023). Tricolor narrowband planar perovskite photodetectors based on FP microcavity structure. Opt. Express.

[105] Liu J.J., Ho W.J., Chiang C.C., Teng C.J., Yu C.C., Li Y.C. (2018). Fabrication and Characterization of Planar-Type Top-Illuminated InP-Based Avalanche Photodetector on Conductive Substrate with Operating Speeds Exceeding 10 Gbps. Sensors.

[106] Martyniuk P., Wang P., Rogalski A., Gu Y., Jiang R., Wang F., Hu W. (2023). Infrared avalanche photodiodes from bulk to 2D materials. Light. Sci. Appl..

[107] Ng T.N., Wong W.S., Chabinyc M.L., Sambandan S., Street R.A. (2008). Flexible image sensor array with bulk heterojunction organic photodiode. Appl. Phys. Lett..

[108] Eckstein R., Strobel N., Rödlmeier T., Glaser K., Lemmer U., Hernandez-Sosa G. (2018). Fully digitally printed image sensor based on organic photodiodes. Adv. Opt. Mater..

[109] Song Y., Zhong Z., He P., Yu G., Xue Q., Lan L., Huang F. (2022). Doping Compensation Enables High-Detectivity Infrared Organic Photodiodes for Image Sensing. Adv. Mater..

[110] Calvi S., Rapisarda M., Valletta A., Scagliotti M., De Rosa S., Tortora L., Branchini P., Mariucci L. (2022). Highly sensitive organic phototransistor for flexible optical detector arrays. Org. Electron..

[111] Rand B.P., Xue J., Lange M., Forrest S.R. (2003). Thin-film organic position sensitive detectors. IEEE Photonics Technol. Lett..

[112] Cabanillas-Gonzalez J., Peña-Rodríguez O., Suarez Lopez I., Schmidt M., Alonso M.I., Goni A.R., Campoy-Quiles M. (2011). Organic position sensitive photodetectors based on lateral donor-acceptor concentration gradients. Appl. Phys. Lett..

[113] Li N., Li Y., Cheng Z., Liu Y., Dai Y., Kang S., Li S., Shan N., Wai S., Ziaja A. (2023). Bioadhesive polymer semiconductors and transistors for intimate biointerfaces. Science.

[114] Mariello M., Kim K., Wu K., Lacour S.P., Leterrier Y. (2022). Recent advances in encapsulation of flexible bioelectronic implants: Materials, technologies, and characterization methods. Adv. Mater..

[115] Wu S.J., Zhao X. (2023). Tissue adhesive semiconductors. Science.

[116] Ferguson J.E., Redish A.D. (2011). Wireless communication with implanted medical devices using the conductive properties of the body. Expert Rev. Med. Devices.

[117] Khan A.N., Cha Y.O., Giddens H., Hao Y. (2022). Recent advances in organ specific wireless bioelectronic devices: Perspective on biotelemetry and power transfer using antenna systems. Engineering.

[118] Tian X., Zeng Q., Kurt S.A., Li R.R., Nguyen D.T., Xiong Z., Li Z., Yang X., Xiao X., Wu C. (2023). Implant-to-implant wireless networking with metamaterial textiles. Nat. Commun..

[119] Dimov I.B., Moser M., Malliaras G.G., McCulloch I. (2022). Semiconducting polymers for neural applications. Chem. Rev..

[120] Liu C., Li Y., Minari T., Takimiya K., Tsukagoshi K. (2012). Forming semiconductor/dielectric double layers by one-step spin-coating for enhancing the performance of organic field-effect transistors. Org. Electron..

[121] Xie Q., Wang L., Zhu Y., Sun Q., Wang L. (2019). Highly sensitive NO_2_ sensors based on organic field effect transistors with Al_2_O_3_/PMMA bilayer dielectrics by sol-spin coating. Org. Electron..

[122] Cho H., Lee H.N., Jeong Y.C., Park Y.M., Kang K.T., Cho K.H. (2020). Solution and evaporation hybrid approach to enhance the stability and pattern resolution characteristics of organic light-emitting diodes. ACS Appl. Mater. Interfaces.

[123] Jung S.W., Kim K.S., Park H.U., Lampande R., Kim S.K., Kim J.H., Han C.W., Choi H.C., Kwon J.H. (2021). Patternable semi-transparent cathode using thermal evaporation for OLED display applications. Adv. Electron. Mater..

[124] DeFranco J.A., Schmidt B.S., Lipson M., Malliaras G.G. (2006). Photolithographic patterning of organic electronic materials. Org. Electron..

[125] Khodagholy D., Doublet T., Gurfinkel M., Quilichini P., Ismailova E., Leleux P., Herve T., Sanaur S., Bernard C., Malliaras G.G. (2011). Highly conformable conducting polymer electrodes for in vivo recordings. Adv. Mater..

[126] Sessolo M., Khodagholy D., Rivnay J., Maddalena F., Gleyzes M., Steidl E., Buisson B., Malliaras G.G. (2013). Easy-to-fabricate conducting polymer microelectrode arrays. Adv. Mater..

[127] Nawaz A., Liu Q., Leong W.L., Fairfull-Smith K.E., Sonar P. (2021). Organic electrochemical transistors for in vivo bioelectronics. Adv. Mater..

[128] Setti L., Fraleoni-Morgera A., Mencarelli I., Filippini A., Ballarin B., Di Biase M. (2007). An HRP-based amperometric biosensor fabricated by thermal inkjet printing. Sens. Actuators B Chem..

[129] Weng B., Liu X., Shepherd R., Wallace G.G. (2012). Inkjet printed polypyrrole/collagen scaffold: A combination of spatial control and electrical stimulation of PC12 cells. Synth. Met..

[130] Greco F., Zucca A., Taccola S., Mazzolai B., Mattoli V. (2013). Patterned free-standing conductive nanofilms for ultraconformable circuits and smart interfaces. ACS Appl. Mater. Interfaces.

[131] Kumar S., Bhushan P., Pandey M., Bhattacharya S. (2019). Additive manufacturing as an emerging technology for fabrication of microelectromechanical systems (MEMS). J. Micromanuf..

[132] Zhang Y., Shi G., Qin J., Lowe S.E., Zhang S., Zhao H., Zhong Y.L. (2019). Recent progress of direct ink writing of electronic components for advanced wearable devices. ACS Appl. Electron. Mater..

[133] Sreenilayam S.P., Ahad I.U., Nicolosi V., Garzon V.A., Brabazon D. (2020). Advanced materials of printed wearables for physiological parameter monitoring. Mater. Today.

[134] Saadi M., Maguire A., Pottackal N.T., Thakur M.S.H., Ikram M.M., Hart A.J., Ajayan P.M., Rahman M.M. (2022). Direct ink writing: A 3D printing technology for diverse materials. Adv. Mater..

[135] Tay R.Y., Song Y., Yao D.R., Gao W. (2023). Direct-ink-writing 3D-printed bioelectronics. Mater. Today.

[136] Choi J.W., Nam Y.S., Lee W.H. (2002). Bioelectronic device consisting of self-assembled biomolecules. Curr. Appl. Phys..

[137] Iost R.M., Crespilho F.N. (2012). Layer-by-layer self-assembly and electrochemistry: Applications in biosensing and bioelectronics. Biosens. Bioelectron..

[138] Wang M., Wang X., Moni P., Liu A., Kim D.H., Jo W.J., Sojoudi H., Gleason K.K. (2017). CVD polymers for devices and device fabrication. Adv. Mater..

[139] Heydari Gharahcheshmeh M., Gleason K.K. (2019). Device fabrication based on oxidative chemical vapor deposition (oCVD) synthesis of conducting polymers and related conjugated organic materials. Adv. Mater. Interfaces.

[140] Torricelli F., Adrahtas D.Z., Bao Z., Berggren M., Biscarini F., Bonfiglio A., Bortolotti C.A., Frisbie C.D., Macchia E., Malliaras G.G. (2021). Electrolyte-gated transistors for enhanced performance bioelectronics. Nat. Rev. Methods Primers.

[141] Wang Z., Bai H., Yu W., Gao Z., Chen W., Yang Z., Zhu C., Huang Y., Lv F., Wang S. (2022). Flexible bioelectronic device fabricated by conductive polymer–based living material. Sci. Adv..

[142] Balakrishnan G., Song J., Mou C., Bettinger C.J. (2022). Recent progress in materials chemistry to advance flexible bioelectronics in medicine. Adv. Mater..

[143] Schwartz D.K. (2001). Mechanisms and kinetics of self-assembled monolayer formation. Annu. Rev. Phys. Chem..

[144] Kwon Y.s., Iwamoto M., Lee T. (2010). Nanoscale Interface for Organic Electronics.

[145] Dai F., Liu X., Yang T., Qian J., Li Y., Gao Y., Xiong P., Ou H., Wu J., Kanehara M. (2019). Fabrication of two-dimensional crystalline organic films by tilted spin coating for high-performance organic field-effect transistors. ACS Appl. Mater. Interfaces.

[146] Gablech I., Głowacki E.D. (2023). State-of-the-Art Electronic Materials for Thin Films in Bioelectronics. Adv. Electron. Mater..

[147] Wang S., Zhao X., Tong Y., Tang Q., Liu Y. (2020). Directly Spin Coating a Low-Viscosity Organic Semiconductor Solution onto Hydrophobic Surfaces: Toward High-Performance Solution-Processable Organic Transistors. Adv. Mater. Interfaces.

[148] Chakraborty A., Herrera D., Fallen P., Hall D., Bampton N., Olivero T., Orlowski M. (2023). Conductive organic electrodes for flexible electronic devices. Sci. Rep..

[149] Ouyang S., Xie Y., Wang D., Zhu D., Xu X., Tan T., Fong H.H. (2015). Surface patterning of PEDOT: PSS by photolithography for organic electronic devices. J. Nanomater..

[150] Dadras-Toussi O., Khorrami M., Louis Sam Titus A.S.C., Majd S., Mohan C., Abidian M.R. (2022). Multiphoton lithography of organic semiconductor devices for 3D printing of flexible electronic circuits, biosensors, and bioelectronics. Adv. Mater..

[151] Son J., Shin H.Y., Choi Y.M., Chae S.G., Park C., Jung B.J., Lee J.K. (2020). Descumming fluorous solution for photolithographic patterning of organic light-emitting diodes. Microelectron. Eng..

[152] Donthu S., Pan Z., Myers B., Shekhawat G., Wu N., Dravid V. (2005). Facile scheme for fabricating solid-state nanostructures using e-beam lithography and solution precursors. Nano Lett..

[153] Gomez N., Lee J.Y., Nickels J.D., Schmidt C.E. (2007). Micropatterned polypyrrole: A combination of electrical and topographical characteristics for the stimulation of cells. Adv. Funct. Mater..

[154] Scholten K., Meng E. (2016). Electron-beam lithography for polymer bioMEMS with submicron features. Microsyst. Nanoeng..

[155] Kolodziej C.M., Maynard H.D. (2012). Electron-beam lithography for patterning biomolecules at the micron and nanometer scale. Chem. Mater..

[156] Lim J.H., Mirkin C.A. (2002). Electrostatically driven dip-pen nanolithography of conducting polymers. Adv. Mater..

[157] Tang Q., Shi S.Q., Huang H., Zhou L.M. (2004). Fabrication of highly oriented microstructures and nanostructures of ferroelectric P (VDF-TrFE) copolymer via dip-pen nanolithography. Superlattices Microstruct..

[158] Eom S.H., Senthilarasu S., Uthirakumar P., Yoon S.C., Lim J., Lee C., Lim H.S., Lee J., Lee S.H. (2009). Polymer solar cells based on inkjet-printed PEDOT: PSS layer. Org. Electron..

[159] Park S., Yang G., Madduri N., Abidian M.R., Majd S. (2014). Hydrogel-Mediated Direct Patterning of Conducting Polymer Films with Multiple Surface Chemistries. Adv. Mater..

[160] Ricoult S.G., Sanati Nezhad A., Knapp-Mohammady M., Kennedy T.E., Juncker D. (2014). Humidified microcontact printing of proteins: Universal patterning of proteins on both low and high energy surfaces. Langmuir.

[161] Hager R., Forsich C., Duchoslav J., Burgstaller C., Stifter D., Weghuber J., Lanzerstorfer P. (2022). Microcontact Printing of Biomolecules on Various Polymeric Substrates: Limitations and Applicability for Fluorescence Microscopy and Subcellular Micropatterning Assays. ACS Appl. Polym. Mater..

[162] Zschieschang U., Halik M., Klauk H. (2008). Microcontact-printed self-assembled monolayers as ultrathin gate dielectrics in organic thin-film transistors and complementary circuits. Langmuir.

[163] Hirata I., Zschieschang U., Yokota T., Kuribara K., Kaltenbrunner M., Klauk H., Sekitani T., Someya T. (2015). High-resolution spatial control of the threshold voltage of organic transistors by microcontact printing of alkyl and fluoroalkylphosphonic acid self-assembled monolayers. Org. Electron..

[164] Liu H., Xie Y., Liu J., Moon K.S., Lu L., Lin Z., Yuan W., Shen C., Zang X., Lin L. (2020). Laser-induced and KOH-activated 3D graphene: A flexible activated electrode fabricated via direct laser writing for in-plane micro-supercapacitors. Chem. Eng. J..

[165] Zhu X., Lin L., Wu R., Zhu Y., Sheng Y., Nie P., Liu P., Xu L., Wen Y. (2021). Portable wireless intelligent sensing of ultra-trace phytoregulator *α*-naphthalene acetic acid using self-assembled phosphorene/Ti3C2-MXene nanohybrid with high ambient stability on laser induced porous graphene as nanozyme flexible electrode. Biosens. Bioelectron..

[166] Yuk H., Lu B., Lin S., Qu K., Xu J., Luo J., Zhao X. (2020). 3D printing of conducting polymers. Nat. Commun..

[167] Plog J., Wang X., Lichade K.M., Pan Y., Yarin A. (2023). Extremely-Fast Electrostatically-Assisted Direct Ink Writing of 2d, 2.5 d and 3d Functional Traces of Conducting Polymer Poly (3, 4-Ethylenedioxythiophene) Polystyrene Sulfonate-Polyethylene Oxide (Pedot: Pss-Peo). J. Colloid Interface Sci..

[168] Lacey S.D., Kirsch D.J., Li Y., Morgenstern J.T., Zarket B.C., Yao Y., Dai J., Garcia L.Q., Liu B., Gao T. (2018). Extrusion-based 3D printing of hierarchically porous advanced battery electrodes. Adv. Mater..

[169] Kee S., Zhang P., Travas-Sejdic J. (2020). Direct writing of 3D conjugated polymer micro/nanostructures for organic electronics and bioelectronics. Polym. Chem..

[170] Won P., Valentine C.S., Zadan M., Pan C., Vinciguerra M., Patel D.K., Ko S.H., Walker L.M., Majidi C. (2022). 3D printing of liquid metal embedded elastomers for soft thermal and electrical materials. ACS Appl. Mater. Interfaces.

[171] Wang Y., Willenbacher N. (2022). Phase-Change-Enabled, Rapid, High-Resolution Direct Ink Writing of Soft Silicone. Adv. Mater..

[172] Tang X., Zhou H., Cai Z., Cheng D., He P., Xie P., Zhang D., Fan T. (2018). Generalized 3D printing of graphene-based mixed-dimensional hybrid aerogels. ACS Nano.

[173] Khlyustova A., Cheng Y., Yang R. (2020). Vapor-deposited functional polymer thin films in biological applications. J. Mater. Chem. B.

[174] Wang X., Zhang X., Sun L., Lee D., Lee S., Wang M., Zhao J., Shao-Horn Y., Dincă M., Palacios T. (2018). High electrical conductivity and carrier mobility in oCVD PEDOT thin films by engineered crystallization and acid treatment. Sci. Adv..

[175] Macchia E., Picca R.A., Manoli K., Di Franco C., Blasi D., Sarcina L., Ditaranto N., Cioffi N., Österbacka R., Scamarcio G. (2020). About the amplification factors in organic bioelectronic sensors. Mater. Horiz..

[176] Naresh V., Lee N. (2021). A review on biosensors and recent development of nanostructured materials-enabled biosensors. Sensors.

[177] Grieshaber D., MacKenzie R., Vörös J., Reimhult E. (2008). Electrochemical biosensors-sensor principles and architectures. Sensors.

[178] Ronkainen N.J., Halsall H.B., Heineman W.R. (2010). Electrochemical biosensors. Chem. Soc. Rev..

[179] Cui L., Wu J., Ju H. (2015). Electrochemical sensing of heavy metal ions with inorganic, organic and bio-materials. Biosens. Bioelectron..

[180] Shanbhag M.M., Manasa G., Mascarenhas R.J., Mondal K., Shetti N.P. (2023). Fundamentals of bio-electrochemical sensing. Chem. Eng. J. Adv..

[181] Wu J., Liu H., Chen W., Ma B., Ju H. (2023). Device integration of electrochemical biosensors. Nat. Rev. Bioeng..

[182] Ozdemir M.S., Marczak M., Bohets H., Bonroy K., Roymans D., Stuyver L., Vanhoutte K., Pawlak M., Bakker E. (2013). A label-free potentiometric sensor principle for the detection of antibody–antigen interactions. Anal. Chem..

[183] Chen C., Wang J. (2020). Optical biosensors: An exhaustive and comprehensive review. Analyst.

[184] Singh A.K., Mittal S., Das M., Saharia A., Tiwari M. (2023). Optical biosensors: A decade in review. Alex. Eng. J..

[185] Damborskỳ P., Švitel J., Katrlík J. (2016). Optical biosensors. Essays Biochem..

[186] Cooper M.A. (2002). Optical biosensors in drug discovery. Nat. Rev. Drug Discov..

[187] Liang X., Li N., Zhang R., Yin P., Zhang C., Yang N., Liang K., Kong B. (2021). Carbon-based SERS biosensor: From substrate design to sensing and bioapplication. NPG Asia Mater..

[188] Borisov S.M., Wolfbeis O.S. (2008). Optical biosensors. Chem. Rev..

[189] Khani S., Hayati M. (2022). Optical biosensors using plasmonic and photonic crystal band-gap structures for the detection of basal cell cancer. Sci. Rep..

[190] Chen Y.T., Lee Y.C., Lai Y.H., Lim J.C., Huang N.T., Lin C.T., Huang J.J. (2020). Review of integrated optical biosensors for point-of-care applications. Biosensors.

[191] Kozma P., Kehl F., Ehrentreich-Förster E., Stamm C., Bier F.F. (2014). Integrated planar optical waveguide interferometer biosensors: A comparative review. Biosens. Bioelectron..

[192] Skládal P. (2016). Piezoelectric biosensors. TrAC Trends Anal. Chem..

[193] Pohanka M. (2018). Overview of piezoelectric biosensors, immunosensors and DNA sensors and their applications. Materials.

[194] Narita F., Wang Z., Kurita H., Li Z., Shi Y., Jia Y., Soutis C. (2021). A review of piezoelectric and magnetostrictive biosensor materials for detection of COVID-19 and other viruses. Adv. Mater..

[195] Koklu A., Ohayon D., Wustoni S., Druet V., Saleh A., Inal S. (2021). Organic bioelectronic devices for metabolite sensing. Chem. Rev..

[196] Bhalla N., Jolly P., Formisano N., Estrela P. (2016). Introduction to biosensors. Essays in Biochemistry.

[197] Coles L., Oluwasanya P.W., Karam N., Proctor C.M. (2022). Fluidic enabled bioelectronic implants: Opportunities and challenges. J. Mater. Chem. B.

[198] Macchia E., Manoli K., Di Franco C., Picca R.A., Österbacka R., Palazzo G., Torricelli F., Scamarcio G., Torsi L. (2020). Organic field-effect transistor platform for label-free, single-molecule detection of genomic biomarkers. ACS Sens..

[199] Wang L., Xie H., Lin Y., Wang M., Sha L., Yu X., Yang J., Zhao J., Li G. (2022). Covalent organic frameworks (COFs)-based biosensors for the assay of disease biomarkers with clinical applications. Biosens. Bioelectron..

[200] Parkula V., Berto M., Diacci C., Patrahau B., Di Lauro M., Kovtun A., Liscio A., Sensi M., Samorì P., Greco P. (2020). Harnessing selectivity and sensitivity in electronic biosensing: A novel lab-on-chip multigate organic transistor. Anal. Chem..

[201] Macchia E., Kovács-Vajna Z.M., Loconsole D., Sarcina L., Redolfi M., Chironna M., Torricelli F., Torsi L. (2022). A handheld intelligent single-molecule binary bioelectronic system for fast and reliable immunometric point-of-care testing. Sci. Adv..

[202] Zhang J., Lan T., Lu Y. (2022). Overcoming major barriers to developing successful sensors for practical applications using functional nucleic acids. Annu. Rev. Anal. Chem..

[203] Bai H., Wang Y., Li X., Guo J. (2023). Electrochemical nucleic acid sensors: Competent pathways for mobile molecular diagnostics. Biosens. Bioelectron..

[204] Fang Y., Li X., Fang Y. (2015). Organic bioelectronics for neural interfaces. J. Mater. Chem. C.

[205] Lee G., Does M.D., Avila R., Kang J., Harkins K.D., Wu Y., Banks W.E., Park M., Lu D., Yan X. (2023). Implantable, Bioresorbable Radio Frequency Resonant Circuits for Magnetic Resonance Imaging. Adv. Sci..

[206] Cheng J., Sheldon E.L., Wu L., Heller M.J., O’Connell J.P. (1998). Isolation of cultured cervical carcinoma cells mixed with peripheral blood cells on a bioelectronic chip. Anal. Chem..

[207] Hsiao Y.S., Yen S.C., Wu P.I., Quinones E.D., Hung S.T., Chen C.S., Tsai S.M. (2022). Microfluidic organic bioelectronic chips for efficient isolation of trophoblast cells using a combination of rational catenation and electrically controllable refining. Mater. Chem. Phys..

[208] Hsiao Y.S., Quiñones E.D., Yen S.C., Yu J., Fang J.T., Chen P., Juang R.S. (2023). PEDOT: PSS-Based Bioelectrodes for Multifunctional Drug Release and Electric Cell-Substrate Impedance Sensing. ACS Appl. Mater. Interfaces.

[209] Deng Y., Qi H., Ma Y., Liu S., Zhao M., Guo Z., Jie Y., Zheng R., Jing J., Chen K. (2022). A flexible and highly sensitive organic electrochemical transistor-based biosensor for continuous and wireless nitric oxide detection. Proc. Natl. Acad. Sci. USA.

[210] Tang W., Fu Y., Huang Y., Li Y., Song Y., Xi X., Yu Y., Su Y., Yan F., Guo X. (2022). Solution processed low power organic field-effect transistor bio-chemical sensor of high transconductance efficiency. NPJ Flex. Electron..

[211] Chen J.C., Kan P., Yu Z., Alrashdan F., Garcia R., Singer A., Lai C.E., Avants B., Crosby S., Li Z. (2022). A wireless millimetric magnetoelectric implant for the endovascular stimulation of peripheral nerves. Nat. Biomed. Eng..

[212] Shafiee H., Lidstone E.A., Jahangir M., Inci F., Hanhauser E., Henrich T.J., Kuritzkes D.R., Cunningham B.T., Demirci U. (2014). Nanostructured optical photonic crystal biosensor for HIV viral load measurement. Sci. Rep..

[213] Lin B., Wang M., Zhao C., Wang S., Chen K., Li X., Long Z., Zhao C., Song X., Yan S. (2022). Flexible organic integrated electronics for self-powered multiplexed ocular monitoring. NPJ Flex. Electron..

[214] Kim S., Baek S., Sluyter R., Konstantinov K., Kim J.H., Kim S., Kim Y.H. (2023). Wearable and implantable bioelectronics as eco-friendly and patient-friendly integrated nanoarchitectonics for next-generation smart healthcare technology. EcoMat.

[215] Nathan V., Jafari R. (2017). Particle filtering and sensor fusion for robust heart rate monitoring using wearable sensors. IEEE J. Biomed. Health Inform..

[216] Kireev D., Sel K., Ibrahim B., Kumar N., Akbari A., Jafari R., Akinwande D. (2022). Continuous cuffless monitoring of arterial blood pressure via graphene bioimpedance tattoos. Nat. Nanotechnol..

[217] Kano S., Kim K., Fujii M. (2017). Fast-response and flexible nanocrystal-based humidity sensor for monitoring human respiration and water evaporation on skin. ACS Sens..

[218] Roy K., Ghosh S.K., Sultana A., Garain S., Xie M., Bowen C.R., Henkel K., Schmeiβer D., Mandal D. (2019). A self-powered wearable pressure sensor and pyroelectric breathing sensor based on GO interfaced PVDF nanofibers. ACS Appl. Nano Mater..

[219] Trung T.Q., Dang T.M.L., Ramasundaram S., Toi P.T., Park S.Y., Lee N.E. (2018). A stretchable strain-insensitive temperature sensor based on free-standing elastomeric composite fibers for on-body monitoring of skin temperature. ACS Appl. Mater. Interfaces.

[220] Yang T.H., Kim J.U., Kim Y.M., Koo J.H., Woo S.Y. (2019). A new blood pulsation simulator platform incorporating cardiovascular physiology for evaluating radial pulse waveform. J. Healthc. Eng..

[221] Karpova E.V., Shcherbacheva E.V., Galushin A.A., Vokhmyanina D.V., Karyakina E.E., Karyakin A.A. (2019). Noninvasive diabetes monitoring through continuous analysis of sweat using flow-through glucose biosensor. Anal. Chem..

[222] Nyein H.Y.Y., Gao W., Shahpar Z., Emaminejad S., Challa S., Chen K., Fahad H.M., Tai L.C., Ota H., Davis R.W. (2016). A wearable electrochemical platform for noninvasive simultaneous monitoring of Ca^2+^ and pH. ACS Nano.

[223] Parlak O., Keene S.T., Marais A., Curto V.F., Salleo A. (2018). Molecularly selective nanoporous membrane-based wearable organic electrochemical device for noninvasive cortisol sensing. Sci. Adv..

[224] Wang C., Shirzaei Sani E., Gao W. (2022). Wearable bioelectronics for chronic wound management. Adv. Funct. Mater..

[225] Shirzaei Sani E., Xu C., Wang C., Song Y., Min J., Tu J., Solomon S.A., Li J., Banks J.L., Armstrong D.G. (2023). A stretchable wireless wearable bioelectronic system for multiplexed monitoring and combination treatment of infected chronic wounds. Sci. Adv..

[226] Campana A., Cramer T., Simon D.T., Berggren M., Biscarini F. (2014). Electrocardiographic recording with conformable organic electrochemical transistor fabricated on resorbable bioscaffold. Adv. Mater..

[227] Ahmed N., Zhu Y. (2020). Early detection of atrial fibrillation based on ECG signals. Bioengineering.

[228] Yang A., Song J., Liu H., Zhao Z., Li L., Yan F. (2023). Wearable Organic Electrochemical Transistor Array for Skin-Surface Electrocardiogram Mapping Above a Human Heart. Adv. Funct. Mater..

[229] Kakria P., Tripathi N., Kitipawang P. (2015). A real-time health monitoring system for remote cardiac patients using smartphone and wearable sensors. Int. J. Telemed. Appl..

[230] Lee Y., Chung J.W., Lee G.H., Kang H., Kim J.Y., Bae C., Yoo H., Jeong S., Cho H., Kang S.G. (2021). Standalone real-time health monitoring patch based on a stretchable organic optoelectronic system. Sci. Adv..

[231] Seesaard T., Wongchoosuk C. (2023). Fabric-based piezoresistive Ti3AlC2/PEDOT: PSS force sensor for wearable E-textile applications. Org. Electron..

[232] Mao P., Li H., Shan X., Davis M., Tang T., Zhang Y., Tong X., Xin Y., Cheng J., Li L. (2023). Stretchable Photodiodes with Polymer-Engineered Semiconductor Nanowires for Wearable Photoplethysmography. ACS Appl. Mater. Interfaces.

[233] Fan Y., Zhang Y., Wu J., Zhao S., Guo J., Wang Z., Chen M., Zhang Q., Li Q. (2023). Free-standing conductive nickel metal-organic framework nanowires as bifunctional electrodes for wearable pressure sensors and Ni-Zn batteries. Iscience.

[234] Yang T., Deng W., Chu X., Wang X., Hu Y., Fan X., Song J., Gao Y., Zhang B., Tian G. (2021). Hierarchically microstructure-bioinspired flexible piezoresistive bioelectronics. ACS Nano.

[235] Jakobs M., Fomenko A., Lozano A.M., Kiening K.L. (2019). Cellular, molecular, and clinical mechanisms of action of deep brain stimulation—A systematic review on established indications and outlook on future developments. EMBO Mol. Med..

[236] Tsopela A., Laborde A., Salvagnac L., Ventalon V., Bedel-Pereira E., Séguy I., Temple-Boyer P., Juneau P., Izquierdo R., Launay J. (2016). Development of a lab-on-chip electrochemical biosensor for water quality analysis based on microalgal photosynthesis. Biosens. Bioelectron..

[237] Cetó X., Voelcker N.H., Prieto-Simón B. (2016). Bioelectronic tongues: New trends and applications in water and food analysis. Biosens. Bioelectron..

[238] Jiang J., Men Y., Pang T., Tang S., Hou Z., Luo M., Sun X., Wu J., Yadav S., Xiong Y. (2023). An integrated supervision framework to safeguard the urban river water quality supported by ICT and models. J. Environ. Manag..

[239] Krkljes D., Kitic G., Petes C., Birgermajer S., Stanojev J., Bajac B., Panic M., Radonic V., Brceski I., Stravs R. (2023). Multiparameter Water Quality Monitoring System for Continuous Monitoring of Fresh Waters. arXiv.

[240] Kashyap B., Kumar R. (2021). Sensing methodologies in agriculture for soil moisture and nutrient monitoring. IEEE Access.

[241] Strand E.J., Bihar E., Gleason S.M., Han S., Schreiber S.W., Renny M.N., Malliaras G.G., McLeod R.R., Whiting G.L. (2022). Printed organic electrochemical transistors for detecting nutrients in whole plant sap. Adv. Electron. Mater..

[242] Fu X., Zheng Z., Sha Z., Cao H., Yuan Q., Yu H., Li Q. (2022). Biorefining waste into nanobiotechnologies can revolutionize sustainable agriculture. Trends Biotechnol..

[243] Alizadeh T., Zare M., Ganjali M.R., Norouzi P., Tavana B. (2010). A new molecularly imprinted polymer (MIP)-based electrochemical sensor for monitoring 2, 4, 6-trinitrotoluene (TNT) in natural waters and soil samples. Biosens. Bioelectron..

[244] Han S., Huang W., Shi W., Yu J. (2014). Performance improvement of organic field-effect transistor ammonia gas sensor using ZnO/PMMA hybrid as dielectric layer. Sens. Actuators B Chem..

[245] Khan M.U., Abbas Y., Abunahla H., Rezeq M., Alazzam A., Alamoodi N., Mohammad B. (2023). Biocompatible Humidity Sensor using Paper Cellulose Fiber/GO Matrix for Human Health and Environment Monitoring. Sens. Actuators B Chem..

[246] Mathur M., Verma A., Singh A., Yadav B., Chaudhary V. (2023). CuMoO4 nanorods-based acetone chemiresistor-enabled non-invasive breathomic-diagnosis of human diabetes and environmental monitoring. Environ. Res..

[247] Scognamiglio V., Arduini F., Palleschi G., Rea G. (2014). Biosensing technology for sustainable food safety. TrAC Trends Anal. Chem..

[248] Rotariu L., Lagarde F., Jaffrezic-Renault N., Bala C. (2016). Electrochemical biosensors for fast detection of food contaminants–trends and perspective. TrAC Trends Anal. Chem..

[249] Curulli A. (2021). Electrochemical biosensors in food safety: Challenges and perspectives. Molecules.

[250] Du T., Huang L., Wang J., Sun J., Zhang W., Wang J. (2021). Luminescent metal-organic frameworks (LMOFs): An emerging sensing platform for food quality and safety control. Trends Food Sci. Technol..

[251] Sharova A.S., Modena F., Luzio A., Melloni F., Cataldi P., Viola F., Lamanna L., Zorn N.F., Sassi M., Ronchi C. (2023). Chitosan-gated organic transistors printed on ethyl cellulose as a versatile platform for edible electronics and bioelectronics. Nanoscale.

[252] Ding N., Dong S., Zhang Y., Lu D., Lin J., Zhao Q., Shi X. (2022). Portable silver-doped prussian blue nanoparticle hydrogels for colorimetric and photothermal monitoring of shrimp and fish freshness. Sens. Actuators B Chem..

[253] Luo X., Huang G., Bai C., Wang C., Yu Y., Tan Y., Tang C., Kong J., Huang J., Li Z. (2023). A versatile platform for colorimetric, fluorescence and photothermal multi-mode glyphosate sensing by carbon dots anchoring ferrocene metal-organic framework nanosheet. J. Hazard. Mater..

[254] Chen Q., Tian R., Liu G., Wen Y., Bian X., Luan D., Wang H., Lai K., Yan J. (2022). Fishing unfunctionalized SERS tags with DNA hydrogel network generated by ligation-rolling circle amplification for simple and ultrasensitive detection of kanamycin. Biosens. Bioelectron..

[255] Li Y., Li N., De Oliveira N., Wang S. (2021). Implantable bioelectronics toward long-term stability and sustainability. Matter.

[256] Shim J.S., Rogers J.A., Kang S.K. (2021). Physically transient electronic materials and devices. Mater. Sci. Eng. R Rep..

[257] Feron K., Lim R., Sherwood C., Keynes A., Brichta A., Dastoor P.C. (2018). Organic bioelectronics: Materials and biocompatibility. Int. J. Mol. Sci..

[258] Song M., Lin X., Peng Z., Xu S., Jin L., Zheng X., Luo H. (2021). Materials and methods of biosensor interfaces with stability. Front. Mater..

[259] Root S.E., Savagatrup S., Printz A.D., Rodriquez D., Lipomi D.J. (2017). Mechanical properties of organic semiconductors for stretchable, highly flexible, and mechanically robust electronics. Chem. Rev..

[260] Yousif E., Haddad R. (2013). Photodegradation and photostabilization of polymers, especially polystyrene. SpringerPlus.

[261] Gray M., Meehan J., Ward C., Langdon S.P., Kunkler I.H., Murray A., Argyle D. (2018). Implantable biosensors and their contribution to the future of precision medicine. Vet. J..

[262] Patel T., Huang J., Krukiewicz K. (2023). Multifunctional organic monolayer-based coatings for implantable biosensors and bioelectronic devices: Review and perspectives. Biosens. Bioelectron. X.

[263] Long Y., Yu Y., Yin X., Li J., Carlos C., Du X., Jiang Y., Wang X. (2019). Effective anti-biofouling enabled by surface electric disturbance from water wave-driven nanogenerator. Nano Energy.

[264] Chen X., Noy A. (2021). Antifouling strategies for protecting bioelectronic devices. APL Mater..

[265] Kumar S., Singh J., Agrawal V., Ahamad M., Malhotra B. (2011). Biocompatible self-assembled monolayer platform based on (3-glycidoxypropyl) trimethoxysilane for total cholesterol estimation. Anal. Methods.

[266] AlKhoury H., Hautmann A., Erdmann F., Zhou G., Stojanović S., Najman S., Groth T. (2020). Study on the potential mechanism of anti-inflammatory activity of covalently immobilized hyaluronan and heparin. J. Biomed. Mater. Res. A.

[267] Fruncillo S., Su X., Liu H., Wong L.S. (2021). Lithographic processes for the scalable fabrication of micro-and nanostructures for biochips and biosensors. ACS Sens..

[268] Bobrowski T., Conzuelo F., Ruff A., Hartmann V., Frank A., Erichsen T., Nowaczyk M.M., Schuhmann W. (2020). Scalable Fabrication of Biophotoelectrodes by Means of Automated Airbrush Spray-Coating. ChemPlusChem.

[269] Xu M., Obodo D., Yadavalli V.K. (2019). The design, fabrication, and applications of flexible biosensing devices. Biosens. Bioelectron..

[270] Xu C., Yang Y., Gao W. (2020). Skin-interfaced sensors in digital medicine: From materials to applications. Matter.

[271] Neshani S., Momeni K., Chen D.J., Neihart N.M. (2023). Highly Sensitive Readout Interface for Real-Time Differential Precision Measurements with Impedance Biosensors. Biosensors.

[272] Lundager K., Zeinali B., Tohidi M., Madsen J.K., Moradi F. (2016). Low power design for future wearable and implantable devices. J. Low Power Electron. Appl..

[273] Song Y., Mukasa D., Zhang H., Gao W. (2021). Self-powered wearable biosensors. Acc. Mater. Res..

[274] Dweiri Y.M., Eggers T., McCallum G., Durand D.M. (2015). Ultra-low noise miniaturized neural amplifier with hardware averaging. J. Neural Eng..

[275] Ramesh M., Janani R., Deepa C., Rajeshkumar L. (2022). Nanotechnology-enabled biosensors: A review of fundamentals, design principles, materials, and applications. Biosensors.

[276] Wang X., Lu D., Liu Y., Wang W., Ren R., Li M., Liu D., Liu Y., Liu Y., Pang G. (2022). Electrochemical signal amplification strategies and their use in olfactory and taste evaluation. Biosensors.

[277] Fischer A.C., Forsberg F., Lapisa M., Bleiker S.J., Stemme G., Roxhed N., Niklaus F. (2015). Integrating mems and ics. Microsyst. Nanoeng..

[278] Zhao Z., Cea C., Gelinas J.N., Khodagholy D. (2021). Responsive manipulation of neural circuit pathology by fully implantable, front-end multiplexed embedded neuroelectronics. Proc. Natl. Acad. Sci. USA.

[279] Stuart T., Hanna J., Gutruf P. (2022). Wearable devices for continuous monitoring of biosignals: Challenges and opportunities. APL Bioeng..

[280] Ruiz-Valdepeńas Montiel V., Sempionatto J.R., Esteban-Fernández de Ávila B., Whitworth A., Campuzano S., Pingarrón J.M., Wang J. (2018). Delayed sensor activation based on transient coatings: Biofouling protection in complex biofluids. J. Am. Chem. Soc..

[281] Xu J., Lee H. (2020). Anti-biofouling strategies for long-term continuous use of implantable biosensors. Chemosensors.

[282] Chan D., Chien J.C., Axpe E., Blankemeier L., Baker S.W., Swaminathan S., Piunova V.A., Zubarev D.Y., Maikawa C.L., Grosskopf A.K. (2022). Combinatorial polyacrylamide hydrogels for preventing biofouling on implantable biosensors. Adv. Mater..

[283] Camara C., Peris-Lopez P., Tapiador J.E. (2015). Security and privacy issues in implantable medical devices: A comprehensive survey. J. Biomed. Inform..

[284] McLamore E.S., Alocilja E., Gomes C., Gunasekaran S., Jenkins D., Datta S.P., Li Y., Mao Y.J., Nugen S.R., Reyes-De-Corcuera J.I. (2021). FEAST of biosensors: Food, environmental and agricultural sensing technologies (FEAST) in North America. Biosens. Bioelectron..

[285] Ibrahim A.U., Al-Turjman F., Sa’id Z., Ozsoz M. (2022). Futuristic CRISPR-based biosensing in the cloud and internet of things era: An overview. Multimed. Tools Appl..

[286] Sathya D., Ganesh Kumar P. (2017). Secured remote health monitoring system. Healthc. Technol. Lett..

[287] Garg N., Wazid M., Das A.K., Singh D.P., Rodrigues J.J., Park Y. (2020). BAKMP-IoMT: Design of blockchain enabled authenticated key management protocol for internet of medical things deployment. IEEE Access.

[288] Zhang J., Huang H., Song G., Huang K., Luo Y., Liu Q., He X., Cheng N. (2022). Intelligent biosensing strategies for rapid detection in food safety: A review. Biosens. Bioelectron..

[289] Manickam P., Mariappan S.A., Murugesan S.M., Hansda S., Kaushik A., Shinde R., Thipperudraswamy S. (2022). Artificial intelligence (AI) and internet of medical things (IoMT) assisted biomedical systems for intelligent healthcare. Biosensors.

[290] Bresnahan P.J., Takeshita Y., Wirth T., Martz T.R., Cyronak T., Albright R., Wolfe K., Warren J.K., Mertz K. (2021). Autonomous in situ calibration of ion-sensitive field effect transistor pH sensors. Limnol. Oceanogr. Methods.

[291] Li X., Wang Y., Yang T., Du Y., Chen Y., Gong D., Zhou Q., Sun X. (2021). Closed-loop control for self-calibration of accelerometer achieved through integrated sensor and actuator system. Microsyst. Technol..

[292] Kang Y., Mouring S., de Clerck A., Mao S., Ng W., Ruan H. (2022). Development of a Flexible Integrated Self-Calibrating MEMS Pressure Sensor Using a Liquid-to-Vapor Phase Change. Sensors.

[293] Vaghasiya J.V., Mayorga-Martinez C.C., Pumera M. (2023). Wearable sensors for telehealth based on emerging materials and nanoarchitectonics. NPJ Flex. Electron..

[294] Granelli R., Alessandri I., Gkoupidenis P., Vassalini I., Kovács-Vajna Z.M., Blom P.W., Torricelli F. (2022). High-Performance Bioelectronic Circuits Integrated on Biodegradable and Compostable Substrates with Fully Printed Mask-Less Organic Electrochemical Transistors. Small.

[295] Bandodkar A.J., You J.M., Kim N.H., Gu Y., Kumar R., Mohan A.V., Kurniawan J., Imani S., Nakagawa T., Parish B. (2017). Soft, stretchable, high power density electronic skin-based biofuel cells for scavenging energy from human sweat. Energy Environ. Sci..

[296] Zou Y., Bo L., Li Z. (2021). Recent progress in human body energy harvesting for smart bioelectronic system. Fundam. Res..

